# METTL3 promotes prostatic hyperplasia by regulating PTEN expression in an m^6^A-YTHDF2-dependent manner

**DOI:** 10.1038/s41419-022-05162-4

**Published:** 2022-08-19

**Authors:** Jiaren Li, Hanyu Yao, Jin Huang, Chao Li, Yichuan Zhang, Ran Xu, Zhenting Wang, Zhi Long, Jin Tang, Long Wang

**Affiliations:** 1 Department of Urology, The Third Xiangya Hospital, Central South University, Changsha, Hunan 410013 China; 2 Department of Urology, The Second Xiangya Hospital, Central South University, Changsha, Hunan 410028 China; 3 Department of Urology, Affiliated Haikou Hospital of Xiangya Medical College, Central South University, Haikou, 570208 Hainan China

**Keywords:** Cell biology, Molecular biology

## Abstract

Uncontrolled epithelial cell proliferation in the prostate transition zone and the hyper-accumulation of mesenchymal-like cells derived from the epithelial-mesenchymal transition (EMT) of prostatic epithelium are two key processes in benign prostatic hyperplasia (BPH). m^6^A RNA modification affects multiple cellular processes, including cell proliferation, apoptosis, and differentiation. In this study, the aberrant up-regulation of methylase METTL3 in BPH samples suggests its potential role in BPH development. Elevated m^6^A modification in the prostate of the BPH rat was partially reduced by METTL3 knockdown. METTL3 knockdown also partially reduced the prostatic epithelial thickness and prostate weight, significantly improved the histological features of the prostate, inhibited epithelial proliferation and EMT, and promoted apoptosis. In vitro, METTL3 knockdown decreased TGF-β-stimulated BPH-1 cell proliferation, m^6^A modification, and EMT, whereas promoted cell apoptosis. METTL3 increased the m^6^A modification of PTEN and inhibited its expression through the reading protein YTHDF2. PTEN knockdown aggravated the molecular, cellular, and pathological alterations in the prostate of BPH rats and amplified TGF-β-induced changes in BPH-1 cells. More importantly, PTEN knockdown partially abolished the improving effects of METTL3 knockdown both in vivo and in vitro. In conclusion, the level of m^6^A modification is elevated in BPH; the METTL3/YTHDF2/PTEN axis disturbs the balance between epithelial proliferation and apoptosis, promotes EMT, and accelerates BPH development in an m^6^A modification-related manner.

## Introduction

Benign prostatic hyperplasia (BPH) is a frequent urinary tract disorder in the elderly male that causes lower urinary tract symptoms [1, 2]. While the specific etiology of BPH is largely unknown, several cellular processes have been reported to be involved in BPH initiation and development. BPH disrupts growth regulation and homeostatic control of proliferative and apoptotic behavior in the periurethral and transition zones, resulting in uncontrolled proliferation and migration of prostatic epithelium and enlargement of the cell proliferating compartment [3, 4]. Furthermore, increased mesenchymal-like cells derived from the prostatic epithelium via epithelial-mesenchymal transition (EMT) also contribute to BPH development [5–7]. Agents or factors that regulate the balance of epithelial proliferation, apoptosis, and EMT might be interesting targets for BPH treatment regimens.

N6-methyladenosine (m^6^A) has been identified as the most frequent and abundant post-transcriptional modification, notably in eukaryotic messenger RNA (mRNA) [8, 9]. In BPH, hypermethylation has been demonstrated. By performing a comprehensive molecular investigation of BPH, including genomic, transcriptomic, and epigenetic profiling, Liu et al. [10] discovered that global hypermethylation is the dominant process in BPH at the epigenetic level. Integrating transcription and methylation signatures identifies two BPH subgroups with distinct clinical characteristics and signaling pathways, which have been validated in two independent cohorts. For instance, in BPH samples, promoter methylation of 14-3-3, a potential tumor suppressor gene involved in cell cycle control and death following DNA damage, is homogeneous [11]. Changes in CpG island methylation at MDR1 occur with a frequency of 71%, which may promote growth [12]. Reportedly, m^6^A modification affects multiple cell behaviors, including proliferation, apoptosis, migration, and EMT [13, 14], which all play a critical role in BPH development. Previous studies have shown that m^6^A regulators such as methyltransferase-like 3/14 (METTL3/14) and YTH domain family members, namely, YTHDC2, YTHDF1, and YTHDF2 played crucial functions in the progress of m^6^A modifications, such as mRNA decay [15], pre-ribosomal RNA processing [16], and so on. These mechanisms were associated with diverse physiological and pathological processes, such as inducing pluripotent stem cells [17], regulating inflammatory response [18], adipogenesis [19]. Among then, YTHDF2, as a member of m^6^A “readers”, played a significant role in multiple diseases, such as cardiac hypertrophy [20] and intervertebral disc degeneration [21]. However, the specific effects of m^6^A modification on BPH and potential mechanisms are still unclear.

This study established the rat BPH model by testosterone injection, and m^6^A modification levels, m^6^A loci, and genes with expression differences and altered m^6^A status were analyzed to identify key methylases and target genes. The in vivo and in vitro effects of identified methylase, the predicted binding between methylase and target gene, and the dynamic effects of the methylase and target gene on the BPH model and TGF-β-stimulated BPH-1 cells were investigated. Collectively, the present study aims to demonstrate the specific effects and the underlying mechanism of critical methylases and target genes in BPH.

## Results

### Methylases altered in rat BPH model and correlated with BPH etiology

**Fig. 1:**
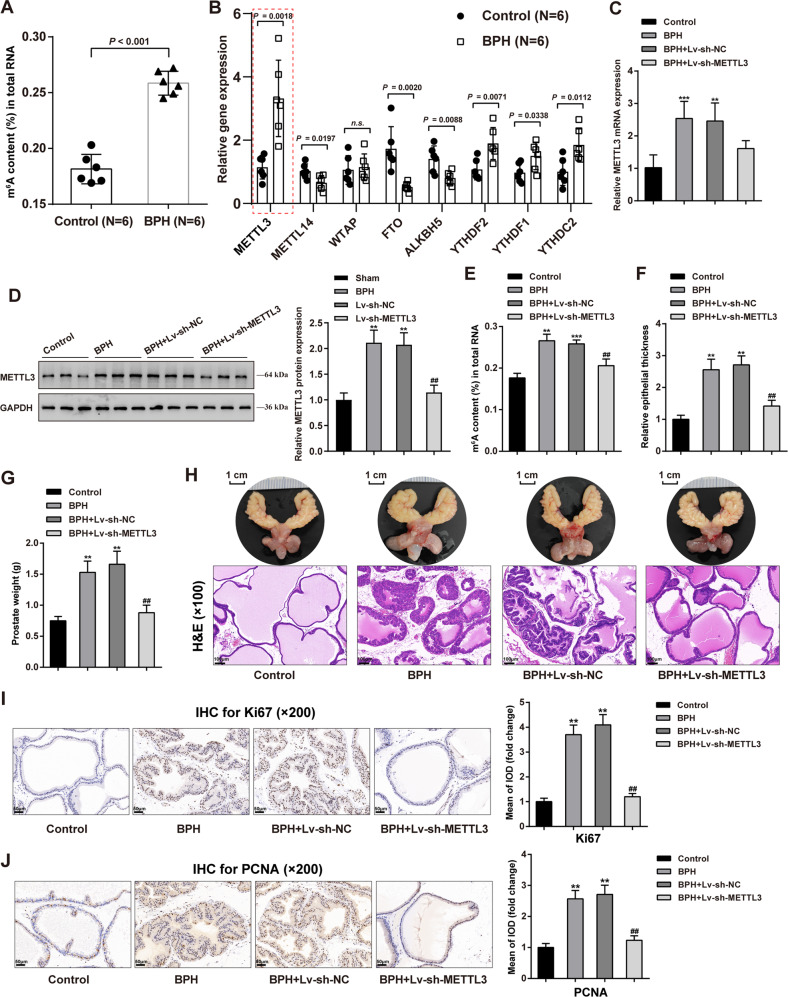
Methylases altered in BPH and methylase METTL3 regulates prostatic hyperplasia in model rats. Prostatic hyperplasia model was induced in rats by subcutaneous injection of testosterone as described in the M&M section. **A** m^6^A status of RNA from normal or hyperplasic rat prostate were determined using an m^6^A RNA Methylation Assay Kit. **B** The expression of methylation-related enzymes was examined in normal and hyperplasic prostate tissues using qRT-PCR, including methylation enzymes: METTL3, METTL14, WTAP; demethylation enzymes: FTO, ALKBH5; reading proteins: YTHDF2, YTHDF1, YTHDC2. **C**, **D** METTL3 knockdown was achieved in rat models by transducing lentivirus containing short hairpin targeting METTL3; METTL3 expression was confirmed in rats in each group using qRT-PCR (**C**) and Immunoblotting (**D**). **E** Methylated N6-methyladenosine (m^6^A) in RNA was evaluated using an m^6^A RNA Methylation Assay Kit. **F**, **G** Prostatic epithelial thickness (**F**) and prostate weight (**G**) were evaluated at the end of the modeling and treatment. **H** A representative prostate with the urinary bladder from each group were shown (upper) and the histopathological characteristics of rats’ prostate from each group were evaluated using H&E staining (down). Scale bar = 1 cm or 100 μm. **I**, **J** The contents of Ki67 and PCNA in rats’ prostate tissues were determined using Immunohistochemical (IHC) staining. Scale bar = 50 μm. **P < 0.01, ***P < 0.001, compared with the control group; ^#^*P* < 0.05, ^##^*P* < 0.01, compared with the BPH + Lv-sh-NC group.

For identifying critical methylases correlated with BPH progression, the m^6^A status of RNA from normal or hyperplasic prostate was determined. Figure 1A shows that m^6^A -modified RNA levels were remarkably higher within hyperplasic prostate samples than in normal rat prostate. The expression levels of methylation-associated enzymes, including methylation enzymes: METTL3 and METTL14; m^6^A methyltransferase complex WTAP; demethylation enzymes: FTO, ALKBH5; and reading proteins: YTHDF2, YTHDF1, YTHDC2 were evaluated. Figure 1B shows that methylase METTL3 expression level was remarkably upregulated within BPH samples (*P* = 0.0018). Considering the role of METTL3 in BPH development remains unclear, METTL3 was selected for further investigations.

### Methylase METTL3 regulates prostatic hyperplasia in model rats

For investigating the specific effects of METTL3 on BPH, the prostatic hyperplasia model was induced in rats by subcutaneous injection of testosterone as described, and METTL3 knockdown was achieved in rat models by injecting lentivirus containing sh-METTL3. METTL3 knockdown was confirmed in rats in each group using qRT-PCR and Immunoblotting (Fig. 1C, D). The levels of m^6^A-modified RNA were evaluated in four groups. Figure 1E shows that the m^6^A modification level was dramatically increased in the BPH and BPH + Lv-sh-NC groups than in normal control (**P < 0.01, ***P < 0.001), whereas remarkably decreased within the BPH + Lv-sh-METTL3 group than the BPH + Lv-sh-NC group (^##^*P* < 0.01). These findings indicate that m^6^A modification increases in BPH rats, but METTL3 knockdown partially reduces m^6^A modification in BPH rats.

Regarding related symptoms and pathological characteristics, the prostatic epithelial thickness and prostate weight increased in BPH rats compared with the control group (**P < 0.01), whereas partially decreased by METTL3 knockdown (^##^*P* < 0.01; Fig. 1F, G). A representative prostate from each group was shown and the histopathological characteristics of rats’ prostates from each group were evaluated using H&E staining. Figure 1H shows that compared with those of the control group, the thickness of the epithelial layer surrounding glands and the stromal components in the rats’ prostate from the BPH and BPH + Lv-sh-NC groups were significantly enhanced, indicating the presence of BPH. In BPH + Lv-sh-METTL3 group, the prostate’s histological characteristics showed to be significantly enhanced, indicating the underlying anti-BPH therapeutic effect of sh-METTL3 on BPH rats.

**Fig. 2:**
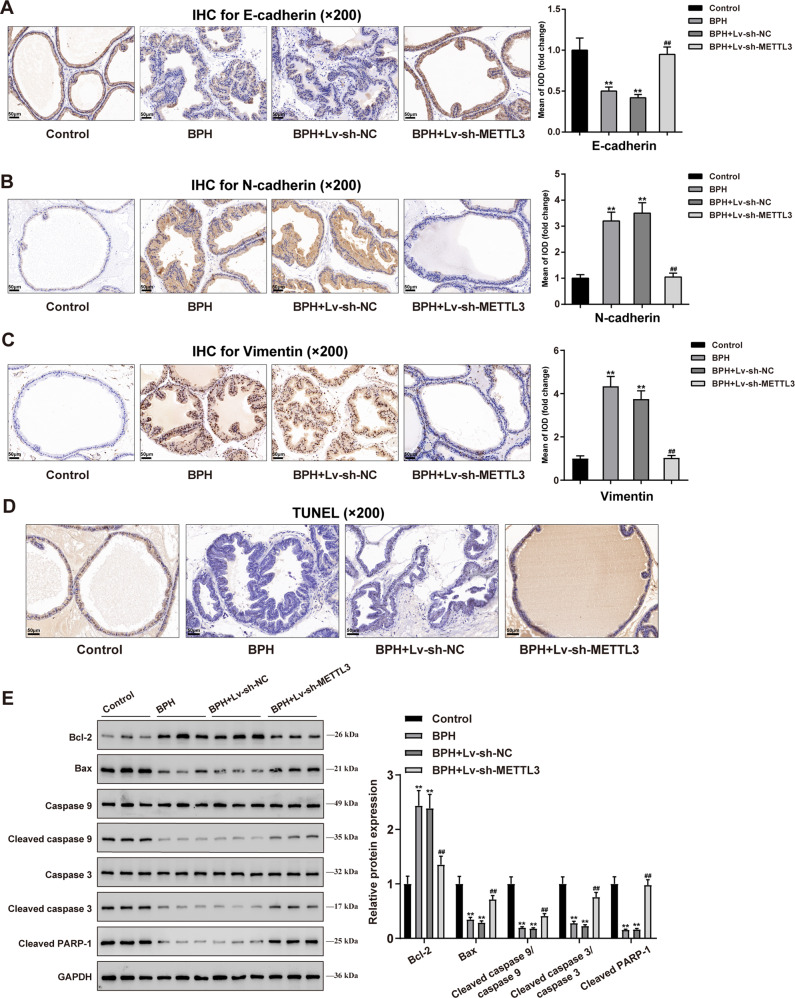
METTL3 knockdown inhibits epithelial-mesenchymal transition (EMT) protein expression and promotes apoptosis in rats’ hyperplasic prostate. **A**–**C** The contents of E-cadherin (**A**), N-cadherin (**B**), and Vimentin (**C**) in rats’ prostate tissues were determined using IHC staining. **D** Cell apoptosis in rats’ prostate tissues was determined using a TUNEL assay. **E** The apoptosis-related proteins (Bcl-2, Bax, Caspase 9, cleaved caspase 9, Caspase 3, cleaved caspase 3, and cleaved PARP-1) levels in prostate tissues in each group were detected by Immunoblotting. All scale bar = 100 μm. **P < 0.01, compared with the control group; ^##^*P* < 0.01, compared with the BPH + Lv-sh-NC group.

Furthermore, IHC staining was employed to determine the contents of Ki67 and PCNA in rats’ prostate tissue samples. Figure 1I, J shows that Ki67 and PCNA levels were dramatically elevated in BPH rats (**P < 0.01) and remarkably decreased by METTL3 knockdown (^##^*P* < 0.01), further suggesting that METTL3 knockdown inhibits cell proliferation in hyperplasic prostate. Moreover, the contents of EMT markers, including E-cadherin, N-cadherin, and vimentin were examined in rats’ prostate tissues. In BPH rats’ prostate, E-cadherin was reduced (**P < 0.01), but N-cadherin and vimentin were elevated (**P < 0.01); after knocking down METTL3, E-cadherin was partially increased, but N-cadherin and vimentin were decreased (^##^*P* < 0.01; Fig. 2A–C), suggesting that METTL3 knockdown attenuates the EMT in BPH rats. Moreover, TUNEL assay results indicated cell apoptosis was significantly inhibited in BPH rats but promoted after METTL3 knockdown (Fig. 2D). And the apoptosis-related proteins (Bcl-2, Bax, Caspase 9, cleaved caspase 9, Caspase 3, cleaved caspase 3, and cleaved PARP-1) levels in prostate tissues of each group were detected by Immunoblotting. In BPH rats, Bcl-2 protein level was significantly increased, while Bax, cleaved caspase 9, cleaved caspase 3 and cleaved PARP-1 protein levels were inhibited. But METTL3 knockdown could reverse these results (Fig. 2E). Considering the changes in Ki67 and PCNA and apoptosis-related proteins, METTL3 might disturb the balance between epithelial proliferation and apoptosis.

### Effects of METTL3 knockdown on TGF-β-treated BPH-1 cell proliferation, apoptosis, and EMT

**Fig. 3:**
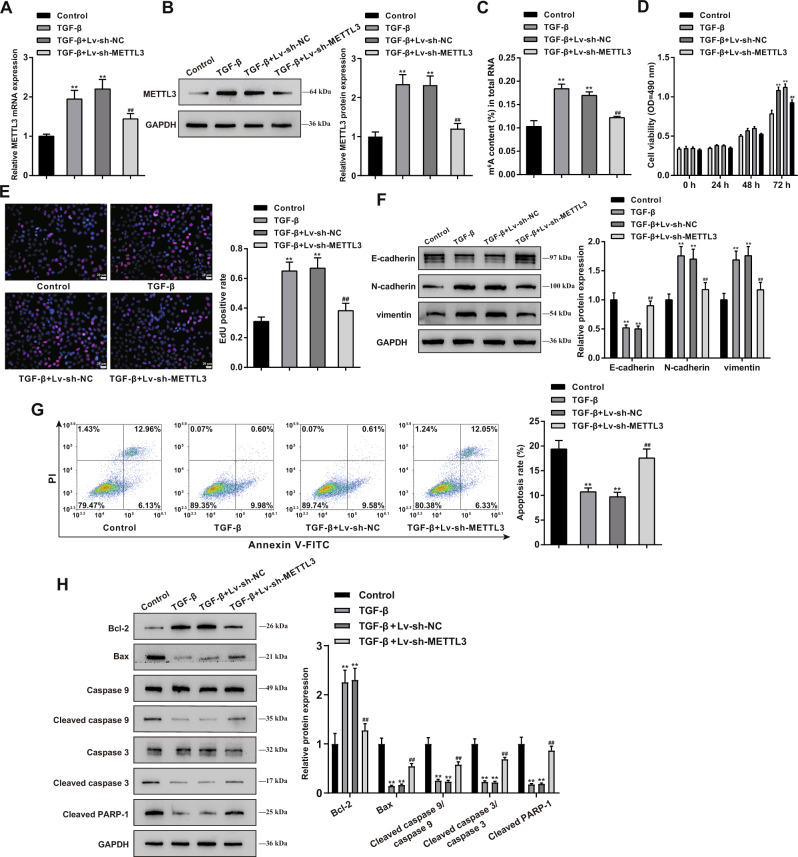
Effects of METTL3 knockdown on proliferation, apoptosis, and EMT of TGF-β-treated BPH-1 cells. METTL3 knockdown was achieved in TGF-β-treated BPH-1 cells by transducing Lv-sh-METTL3 and then examined for **A**, **B** METTL3 expression using qRT-PCR (**A**) and Immunoblotting (**B**); **C** m^6^A status in RNA using an m^6^A RNA Methylation Assay Kit; **D** cell viability using MTT assay; **E** DNA synthesis using EdU assay; Scale bar = 20 μm; **F** the protein levels of E-cadherin, N-cadherin, vimentin using Immunoblotting; **G** cell apoptosis using flow cytometry; **H** the protein levels of Bcl-2, Bax, Caspase 9, cleaved caspase 9, Caspase 3, cleaved caspase 3, and cleaved PARP-1 using Immunoblotting. **P < 0.01, compared with the control group; ^##^*P* < 0.01, compared with the TGF-β + Lv-sh-NC group.

Regarding the cellular effects of METTL3, BPH-1 cells were stimulated with TGF-β for simulating BPH, and METTL3 knockdown was achieved in TGF-β-treated BPH-1 cells by transducing Lv-sh-METTL3. METTL3 knockdown in BPH-1 cells was confirmed using qRT-PCR and Immunoblotting (Fig. 3A, B). Cellular m^6^A status in RNA was examined. Figure 3C shows that TGF-β stimulation resulted in an obvious elevation within m^6^A modification (**P < 0.01), which showed to be partially decreased via METTL3 knockdown (^##^*P* < 0.01). TGF-β treatment also considerably enhanced cell viability (**P < 0.01; Fig. 3D) and DNA synthesis (**P < 0.01; Fig. 3E), which were partially suppressed by METTL3 knockdown (^##^*P* < 0.01; Fig. 3D, E). As for cell EMT, E-cadherin protein contents were reduced, but N-cadherin and vimentin protein contents were elevated in TGF-β-stimulated BPH-1 cells (**P < 0.01); after knocking down METTL3, E-cadherin was elevated, but N-cadherin and vimentin were reduced (^##^*P* < 0.01; Fig. 3F). Regarding cell apoptosis, TGF-β stimulation significantly inhibited cell apoptosis (**P < 0.01), whereas METTL3 knockdown partially promoted cell apoptosis (^##^*P* < 0.01; Fig. 3G). Moreover, TGF-β treatment notably facilitated Bcl-2 protein level and restrained Bax, cleaved caspase 9, cleaved caspase 3, and cleaved PARP-1 protein levels (**P < 0.01); while METTL3 knockdown exerted the opposite effect with TGF-β treatment (^##^*P* < 0.01; Fig. 3H). In addition, the effects of METTL3 knockdown on the cellular survival and apoptosis of the BPH-1 cells under cytotoxic conditions were also determined. As shown in Fig. S1, BPH-1 cells were transduced with sh-METTL3 to achieve METTL3 knockdown and then were stimulated with H_2_O_2_. The results indicated that H_2_O_2_ stimulation markedly inhibited cell viability (Fig. S1A) and enhanced cell apoptosis (Fig. S1B), and METTL3 knockdown further reinforced the effect of H_2_O_2_ treatment on BPH-1 cells.

### PTEN is highly m^6^A-modified and downregulated in BPH

Methylases exert their functions through modified RNAs. For identifying modified RNA correlated with METTL3 functions, three pairs of prostate tissues were collected from normal control rats’ prostates and BPH rats’ prostates for methylation sequencing analysis. From the map showing m^6^A modification distribution in the chromosome region, it can be seen that the vast majority of m^6^A modification loci in the six samples are in the CDS coding region and the 3′-UTR region (Fig. S2A), suggesting the importance of the m^6^A locus in BPH. The proportion chart also saw a similar trend: most m^6^A modification loci were in CDS (56.69%), followed by 3′-UTR (38.15%), and only a very small proportion were in 5′-UTR (5.16%) (Fig. S2B). By comparing the m^6^A peak differences between the control and BPH prostate samples, 35 m^6^A loci belonging to 30 genes were negatively regulated (logFC < 0.25, *P* < 0.05), 12 of which were located in CDS and 22 in 3′-UTR; 257 m^6^A loci belonging to 211 genes were upregulated during BPH (logFC > 1.25, *P* < 0.05), 135 loci in the CDS and 68 loci in the 3′-UTR (Fig. S2C). These 241 altered genes were then applied for functional enrichment annotation. Fig. S3A shows that these 241 genes were enriched in glandular development, differentiation of myeloid leukocytes, skeletal muscle development, and negative regulation of epithelial cell migration, which were correlated with BPH development. Analysis of protein interaction networks and MCODE modules revealed that core genes regulating BPH are associated with cellular stress response, mRNA metabolic processes, and the regulation of protein kinase activity (Fig. S3B).

**Fig. 4:**
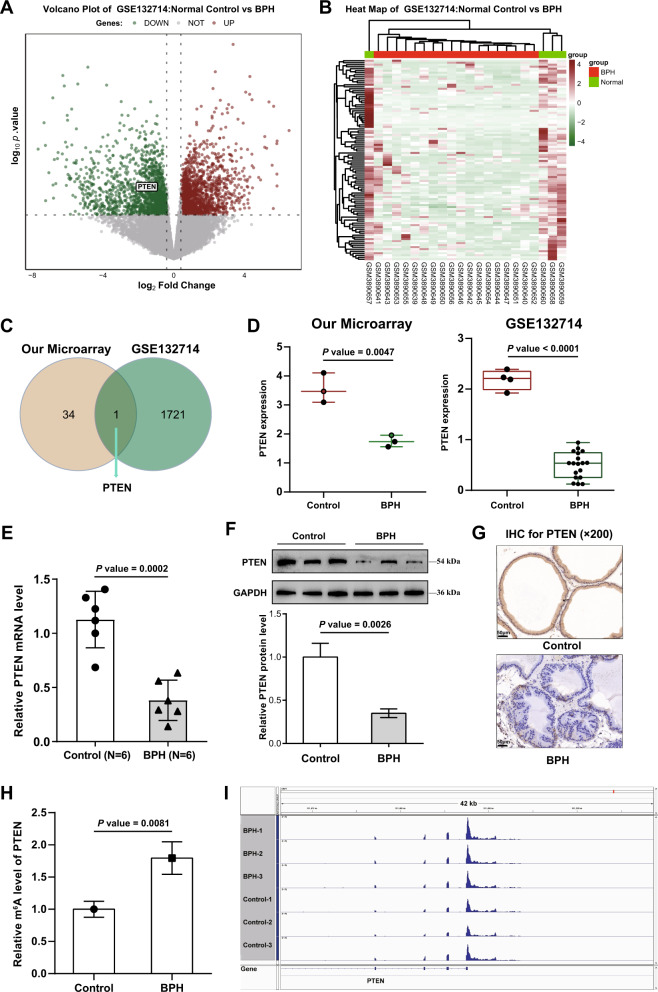
PTEN is highly m6A-modified and downregulated in BPH. **A**, **B** The expression profile between control and BPH samples was analyzed using the R language LIMMA package based on GSE132714. **C** Genes with altered m^6^A modification status and altered expression levels were compared, and PTEN was selected. **D** PTEN expression in normal control and BPH samples according to our microarray and GSE132714. **E** the mRNA level of PTEN in normal control and BPH samples were determined using RT-PCR. **F**, **G** PTEN protein levels in normal control and BPH samples were determined using Immunoblotting and IHC staining. **H** The m^6^A modification status of PTEN in normal control and BPH samples was determined using an m^6^A RNA Methylation Assay Kit. **I** The m^6^A modification status in PTEN 3′UTR was analyzed using IGV software.

Then, the differential genes were cross-screened in combination with the expression profile between control and BPH samples, and genes with changes in m^6^A modification and mRNA expression were analyzed. The expression profile between control and BPH samples was analyzed using the R language LIMMA package based on GSE132714. Figure 4A, B shows that 1722 downregulated genes (logFC < −0.4, *P* < 0.05) and 1677 upregulated genes (logFC > 0.4, *P* < 0.05) were identified, among which PTEN was with increased m^6^A modification status and downregulated expression level (Fig. 4C). According to our methylation sequencing analysis and GSE132714, PTEN mRNA expression showed to be remarkably reduced within BPH samples than the normal controls (Fig. 4D). Consistently, PTEN mRNA and protein contents showed remarkably reduced within BPH samples than in the normal control samples, as inferred from qRT-PCR, Immunoblotting, and IHC staining results (Fig. 4E–G). Importantly, the PTEN m^6^A modification level was dramatically elevated in BPH samples than in the normal control (Fig. 4H). Moreover, most m^6^A modification loci in PTEN were in the 3′-UTR (Fig. 4I), suggesting that elevated m^6^A modification in PTEN might play a role in BPH.

### METTL3 mediates m^6^A modification of PTEN and regulates its expression through reading protein YTHDF2

**Fig. 5:**
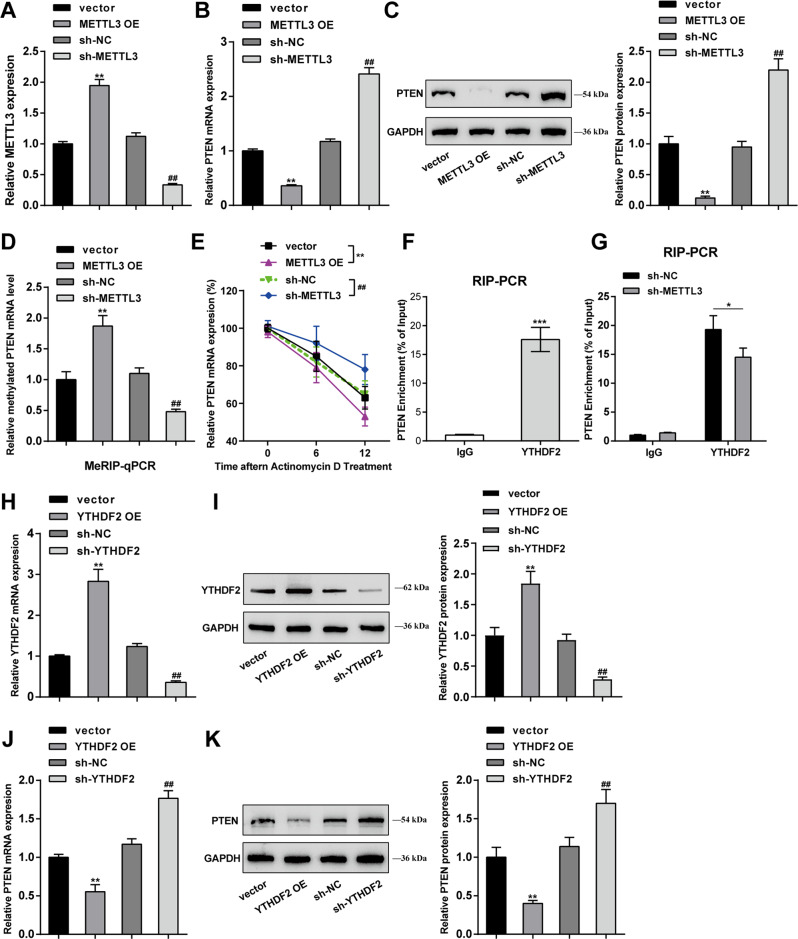
METTL3 mediates m6A modification of PTEN and regulates its expression through reading protein YTHDF2. **A** METTL3 overexpression or knockdown was achieved in BPH-1 cells by transfecting OE-METTL3 or sh-METTL3 and confirmed using qRT-PCR. BPH-1 cells were transduced with OE-METTL3 or sh-METTL3 and examined for PTEN mRNA expression using qRT-PCR (**B**); PTEN protein levels using Immunoblotting (**C**). The m^6^A modification status of PTEN using Me-RIP assay (**D**); PTEN mRNA synthesis using actinomycin D assay (**E**). **P < 0.01, compared with the vector group; ^##^*P* < 0.01, compared with the sh-NC group. **F** The binding between YTHDF2 and PTEN was validated in BPH-1 cells using RIP assay with anti-IgG and anti-YTHDF2. ***P < 0.001, compared with the anti-IgG group. **G** The binding between YTHDF2 and PTEN was validated in sh-NC or sh-METTL3 transduced BPH-1 cells using RIP assay with anti-IgG and anti-YTHDF2. *P < 0.05, compared with the sh-NC group. (**H**, **I**) YTHDF2 overexpression or knockdown was achieved in BPH-1 cells by transfecting OE-YTHDF2 or sh-YTHDF2 and confirmed using qRT-PCR and Immunoblotting, respectively. (**J**, **K**) BPH-1 cells were transduced with OE-YTHDF2 or sh-YTHDF2 and examined for PTEN mRNA expression and protein levels using qRT-PCR and Immunoblotting, respectively. **P < 0.01, compared with the vector group; ^##^*P* < 0.01, compared with the sh-NC group.

Prior to analyzing the specific effects of PTEN upon BPH, the effects and mechanism of METTL3 in regulating PTEN were first explored. OE-METTL3/sh-METTL3 was transfected to achieve METTL3 overexpression/knockdown in BPH-1 cells, as confirmed by qRT-PCR (Fig. 5A). Within BPH-1 cells, METTL3 overexpression dramatically downregulated PTEN mRNA expression and decreased PTEN protein levels, whereas METTL3 knockdown exerted opposing effects on PTEN (**P < 0.01, compared with the vector group; ^##^*P* < 0.01, compared with the sh-NC group; Fig. 5B, C). As for directly investigating the m^6^A modification status of PTEN by METTL3, the Me-RIP assay was performed, and METTL3 promoted the m^6^A modification level of PTEN mRNA (**P < 0.01, compared with the vector group; ^##^*P* < 0.01, compared with the sh-NC group; Fig. 5D). Furthermore, the actinomycin D RNA synthesis assay showed that PTEN mRNA levels were highly unstable in METTL3-overexpressing cells, while METTL3 knockdown showed the opposite effect (**P < 0.01, compared with the vector group; ^##^*P* < 0.01, compared with the sh-NC group; Fig. 5E). Moreover, METTL3-mediated PTEN downregulation has shown to affect the important cancer-associated processes [22, 23], the METTL3 and PTEN expression and their correlation in prostate cancer patients were analyzed based on bioinformatic analysis (Fig. S4). METTL3 expression showed to be dramatically upregulated within prostatic carcinoma samples in comparison with normal control, according to The Cancer Genome Atlas Prostate Adenocarcinoma (TCGA-PRAD) dataset (Fig. S4A) and Gene Expression Omnibus (GEO) dataset (GSE200879) (Fig. S4D). While PTEN expression was observably downregulated in prostatic carcinoma samples when compared to normal control, according to the TCGA-PRAD dataset (Fig. S4B) and GSE200879 dataset (Fig. S4E). Then, Pearson’s correlation analysis based on TCGA-PRAD, and GSE200879 datasets was conducted to analyze the correlation between METTL3 and PTEN expression; according to these two datasets, METTL3 and PTEN were negatively correlated (Fig. S4C, F).

YTHDF2 has been reported that could induce target mRNA degradation by reading m^6^A modification sites [24]. In addition, YTHDF2 was upregulated in BPH model rats (Fig. 1B). To explore the role of YTHDF2 in BPH progress, the effects of YTHDF2 silencing on TGF-β-treated BPH-1 cell proliferation, apoptosis, and EMT were investigated (Fig. S5). Firstly, YTHDF2 knockdown was achieved in TGF-β-treated BPH-1 cells by transducing sh-YTHDF2 and confirmed using qRT-PCR (Fig. S5A). YTHDF2 knockdown observably suppressed TGF-β-treated BPH-1 cell viability (Fig. S5B) and DNA synthesis (Fig. S5C). As for cell EMT, YTHDF2 knockdown promoted E-cadherin protein contents and inhibited N-cadherin and vimentin protein contents (Fig. S5D). Regarding cell apoptosis, YTHDF2 knockdown significantly facilitated cell apoptosis (Fig. S5E) and apoptosis-related protein levels (Fig. S5F). Therefore, the binding between YTHDF2 and PTEN was validated in BPH-1 cells using RIP assay with anti-IgG and anti-YTHDF2. Figure 5F shows that in immunoprecipitate by anti-YTHDF2, the enrichment levels of PTEN were dramatically higher than those in anti-IgG immunoprecipitate (***P < 0.001). Moreover, in immunoprecipitate by anti-YTHDF2 in sh-METTL3 transduced BPH-1 cells, the enrichment levels of PTEN were significantly lower than those in sh-NC transduced BPH-1 cells (*P < 0.05; Fig. 5G), suggesting that METTL3 modulates PTEN expression through YTHDF2. For validating the speculation, OE-YTHDF2/sh-YTHDF2 was transfected to achieve YTHDF2 overexpression/knockdown in BPH-1 cells, as confirmed by qRT-PCR and Immunoblotting (**P < 0.01, compared with the vector group; ^##^*P* < 0.01, compared with the sh-NC group; Fig. 5H, I). In BPH-1 cells, YTHDF2 overexpression also significantly downregulated PTEN mRNA expression and decreased PTEN protein levels, whereas YTHDF2 knockdown exerted opposite effects on PTEN (**P < 0.01, compared with the vector group; ^##^*P* < 0.01, compared with the sh-NC group; Fig. 5J, K).

### METTL3 regulates PTEN and affects cell proliferation and EMT in prostatic hyperplasia model rats

**Fig. 6:**
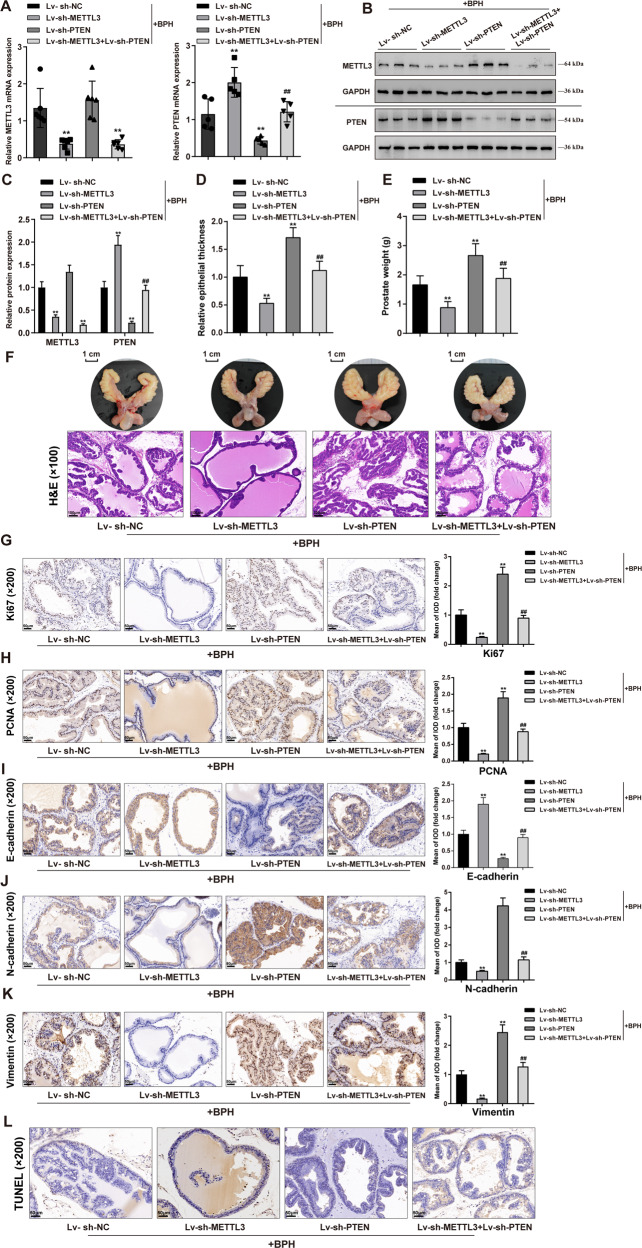
METTL3 regulates PTEN and affects cell proliferation and EMT in prostatic hyperplasia model rats. BPH model was established in rats as described above; rats were injected with Lv-sh-METTL3 and/or Lv-sh-PTEN and examined for **A**–**C** the mRNA and protein expressions of METTL3 and PTEN in rats’ prostate using qRT-PCR and Immunoblotting; **D**–**E** prostatic epithelial thickness and prostate weight at the end of the modeling and treatment; **F** a representative prostate with the urinary bladder from each group (upper) and the histopathological characteristics of rats’ prostate from each group using H&E staining (down); Scale bar = 1 cm or 100 μm; **G**–**K** the contents of Ki67, PCNA, E-cadherin, N-cadherin, and vimentin in rats’ prostate tissues using Immunohistochemical (IHC) staining; **L** cell apoptosis in rats’ prostate using TUNEL assay; scale bar = 50 μm. **P < 0.01, compared with the Lv-sh-NC group. ^##^*P* < 0.01, compared with the Lv-sh-METTL3 group.

After confirming METTL3 regulating PTEN through YTHDF2, the dynamic effects of METTL3 and PTEN on the BPH model and cell model were investigated. BPH rats were injected with Lv-sh-METTL3 and/or Lv-sh-PTEN and examined for the mRNA and protein expressions of METTL3 and PTEN in rats’ prostate; in BPH rats’ prostate, METTL3 mRNA and protein expressions were downregulated by sh-METTL3; while didn’t affect by sh-PTEN (Fig. 6A–C). And, PTEN mRNA and protein expressions were upregulated after knocking down METTL3 but downregulated after Lv-sh-PTEN injection (**P < 0.01), whereas Lv-sh-PTEN significantly reversed the effects of METTL3 knockdown upon PTEN expression (^##^*P* < 0.01; Fig. 6A–C). METTL3 knockdown significantly reduced, whereas PTEN knockdown increased prostatic epithelial thickness and prostate weight (*P* < 0.01), and the effects of METTL3 knockdown were partially eliminated by PTEN knockdown (^##^*P* < 0.01; Fig. 6D, E). A representative prostate from each group was shown (Fig. 6F). H&E staining also indicated the improvement of hyperplasia symptoms by METTL3 knockdown but the exacerbation by PTEN knockdown (*P* < 0.01). Similarly, the improving effects of METTL3 knockdown were partially abolished by PTEN knockdown (^##^*P* < 0.01; Fig. 6F). PTEN knockdown significantly increased the contents of Ki67, PCNA, N-cadherin, and vimentin (*P* < 0.01), but decreased E-cadherin in rats’ prostate tissues and significantly reversed the effects of METTL3 knockdown upon these proteins (^##^*P* < 0.01; Fig. 6G–K). Cell apoptosis investigation saw similar trends: METTL3 knockdown significantly promoted, whereas PTEN knockdown inhibited apoptosis in hyperplasic prostate (*P* < 0.01), and PTEN knockdown partially attenuated the effects of METTL3 on apoptosis (^##^*P* < 0.01; Fig. 6L). Moreover, the dynamic effects of METTL3 and PTEN on the levels of transcription factors orchestrating proliferation, EMT, and apoptosis in BPH model were also investigated (Fig. S6A). METTL3 knockdown significantly inhibited p53, c-Myc, NF-κB, Zeb1, Zeb2, Snail, and Twist1 levels; whereas PTEN knockdown notably promoted p53, c-Myc, NF-κB, Zeb1, Zeb2, Snail, and Twist1 levels, and PTEN knockdown partially attenuated the effects of METTL3 on these transcription factors.

### METTL3 affects TGF-β-stimulated BPH-1 cell phenotype through PTEN

**Fig. 7:**
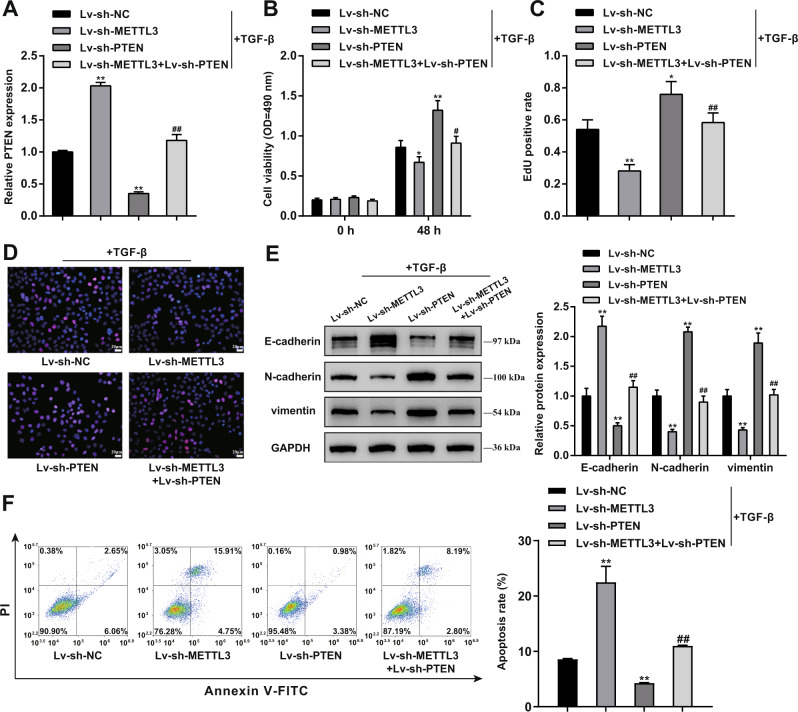
METTL3 affects the cell phenotypes of TGF-β-stimulated. BPH-1 cells through PTEN BPH-1 cells were co-transduced with Lv-sh-METTL3 and Lv-sh-PTEN and examined for PTEN mRNA expression using qRT-PCR (**A**); cell viability using MTT assay (**B**); DNA synthesis using EdU assay (**C**, **D**); Scale bar = 20 μm; the protein levels of E-cadherin, N-cadherin, and vimentin using Immunoblotting (**E**); cell apoptosis by Flow cytometry (**F**). **P < 0.01, compared with the Lv-sh-NC group. ^##^*P* < 0.01, compared with the Lv-sh-METTL3 group.

In vitro, BPH-1 cells were co-transduced with Lv-sh-METTL3 and Lv-sh-PTEN and examined for PTEN mRNA expression. Similar to in vivo results, PTEN expression was upregulated after knocking down METTL3 but downregulated after Lv-sh-PTEN transduction (**P < 0.01), whereas Lv-sh-PTEN significantly reversed the effects of METTL3 knockdown upon PTEN expression (Fig. 7A). Regarding cell phenotypes, METTL3 knockdown suppressed, while PTEN knockdown enhanced cell viability and DNA synthesis (**P < 0.01); PTEN knockdown partially attenuated METTL3 knockdown effects on BPH-1 proliferation (Fig. 7B–D). As for EMT markers, METTL3 knockdown upregulated E-cadherin but downregulated N-cadherin and vimentin, whereas PTEN knockdown exerted opposite effects (**P < 0.01); similarly, PTEN knockdown partially abolished METTL3 knockdown effects on EMT markers (Fig. 7E). In contrast, METTL3 knockdown promoted, whereas PTEN knockdown suppressed cell apoptosis (**P < 0.01); PTEN knockdown also partially eliminated the effects of METTL3 knockdown on cell apoptosis (Fig. 7F). Consistent with in vivo pathological alteration, METTL3 knockdown markedly promoted p53 level and inhibited c-Myc, NF-κB, Zeb1, Zeb2, Snail, and Twist1 levels; while PTEN knockdown exerted opposite effects, and PTEN knockdown partially eliminated the effects of METTL3 on these transcription factors (Fig. S6B).

## Discussion

In this study, methylase METTL3 expression was upregulated in BPH. Elevated m^6^A modification level was observed in BPH rats’ prostate, which could be partially reduced by METTL3 knockdown. In the prostate of BPH rats, METTL3 knockdown also partially reduced the prostatic epithelial thickness and prostate weight, significantly improved the histological features of the prostate, inhibited epithelial proliferation and EMT, and promoted apoptosis. In vitro, METTL3 knockdown decreased TGF-β-stimulated BPH-1 cell proliferation, m^6^A modification, and EMT, while promoted cell apoptosis. Furthermore, in BPH rats, METTL3 increases the m^6^A modification of PTEN and inhibits its expression through the reading protein YTHDF2. PTEN knockdown aggravated the molecular, cellular, and pathological alterations in the prostate of BPH rat and amplified TGF-β-induced changes in BPH-1 cells. Moreover, PTEN knockdown partially abolished the improving effects of METTL3 knockdown both in vivo and in vitro, suggesting METTL3 exerts its functions through PTEN.

Global hypermethylation is the dominant process in BPH at the epigenetic level [10]. For example, methylation of the 5AR2 promoter in some adult prostates is associated with reduced gene expression, which may translate into a slower growth rate of the prostate gland during adulthood [25]. Low or nonexistent expression of 5AR2 in adult human prostate tissues might be due to methylation of the 5AR2 gene promoter. In males with BPH, aging is associated with increased 5AR2 gene promoter methylation and lower protein expression [26]. In males with symptomatic BPH, higher 5-reductase type 2 gene promoter methylation and reduced protein expression are associated with increasing age and BMI [27]. As a result, DNA methylation has been regarded as a tailored medicinal target for the treatment of BPH patients who are resistant to 5-alpha reductase inhibitor medication [28]. However, studies focusing on m^6^A modification in BPH lack. Herein, the m^6^A modification levels were significantly elevated in BPH samples, and m^6^A peaks were enriched around the stop codon, mainly covering CDS and 3′-UTR. Furthermore, signaling and functional enrichment annotation showed that differentially expressed genes with m^6^A loci were enriched in glandular development and negative regulation of epithelial cell migration, which were correlated with the BPH pathway [29, 30]. During BPH progression, growth factors promote the growth of prostatic epithelial and stromal cells as well as an increase in the expression of molecules related to the EMT, including TGF-β, ZEB-1, and Snail, therefore affecting the onset and progression of BPH [31, 32]. Aberrant proliferation of stromal and epithelial cells causes glandular hypertrophy and bladder outlet obstruction [33]. Considering that EMT and the balance between epithelial proliferation and apoptosis are central events during BPH [34, 35], m^6^A modification might act on BPH etiology and pathology by affecting these molecular and cellular events.

Among differentially expressed methylases between BPH and normal samples, METTL3 showed to be the most upregulated in BPH. Besides, its role in BPH remains unclear. Notably, METTL3 has been reported to enhance the proliferation and migration but inhibit the apoptosis of several types of cancer cells [36–38]. In this study, in BPH rats, knocking down METTL3 indeed significantly ameliorated the symptoms in model rats, including reduced prostatic epithelial thickness and prostate weight and improved histological features. In the meantime, METTL3 knockdown significantly reduced the m^6^A modification level, suggesting that METTL3 might act on BPH by affecting m^6^A modification. Regarding cellular alterations in BPH, as aforementioned, the imbalance between epithelial proliferation and apoptosis is one of the most prominent features of BPH [39]. Loss of apoptosis control increases cell proliferation/cell death ratio, leading to BPH [40, 41]. Consistently, METTL3 knockdown inhibited epithelial proliferation but promoted apoptosis in hyperplastic prostate tissues. In vitro, METTL3 knockdown decreased m^6^A modification and cell proliferation and promoted cell apoptosis in TGF-β-stimulated BPH-1 cells, suggesting that METTL3 knockdown partially rectifies the imbalance between epithelial proliferation and apoptosis. More importantly, METTL3 knockdown also inhibited EMT in vivo and in vitro. EMT permits a polarized epithelial cell to assume a mesenchymal phenotype, which has increased resistance to apoptosis, an amplified production of ECM components, and the capacity to migrate and invade [42, 43], therefore contributing to BPH [44, 45]. Upon TGF-β stimulation, activation of several transcription factors, including Snail, ZEB-1, or Smad, leads to the expression of mesenchymal genes [46, 47]. Therefore, METTL3 might also regulate EMT to affect BPH development.

As for the underlying molecular mechanism, METTL3, as the core component, catalyzes methylation sites supplemented with other regulatory proteins [48]. The modified m^6^A sites are recognized and executed by variable readers to produce different functions or events. In this study, PTEN was with increased m^6^A modification status and downregulated expression level in BPH model rats, therefore being identified as the potential target of METTL3 actions. Previously, the reading protein YTHDF2 has been reported to recognize METTL3-mediated m^6^A modified PTEN mRNA and enhanced PTEN [49]. In this study, YTHDF2 also mediates the negative regulation of PTEN by METTL3. Mechanically, METTL3 m^6^A modified PTEN at an m^6^A loci, which later be recognized by the reading protein YTHDF2, leading to the downregulation of PTEN. Interestingly, PTEN is well-known for its anti-tumor functions in several cancers, inhibiting cell proliferation, enhancing apoptosis, and suppressing the EMT of cancer cells [50, 51], indicating that PTEN might mediate the effects of METTL3 on epithelial cell proliferation, apoptosis, and EMT in BPH in an m^6^A modification-related manner. As speculated, PTEN knockdown aggravated the molecular, cellular, and pathological alterations in BPH rats’ prostate and amplified TGF-β-induced changes in BPH-1 cells; more importantly, PTEN knockdown partially abolished the improving effects of METTL3 knockdown both in vivo and in vitro, suggesting that METTL3 exerts its functions in BPH through mediating the m^6^A modification of PTEN.

**Fig. 8:**
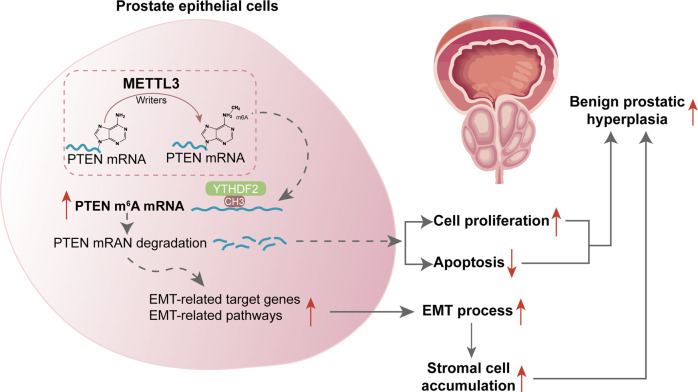
A schematic diagram indicates the mechanisms of how METTL3 promotes prostatic hyperplasia of BPH. The METTL3/YTHDF2/PTEN axis disturbs the balance between epithelial proliferation and apoptosis, promotes EMT, and accelerates BPH development in an m^6^A modification-related manner.

In conclusion, the overall m^6^A modification level is elevated in BPH. The METTL3/YTHDF2/PTEN axis exerts a crucial effect on promoting BPH development by disturbing the balance between epithelial proliferation and apoptosis and promoting EMT (Fig. 8).

## Materials and methods

### Experimental animals and BPH model induction

Mature male Sprague Dawley rats weighing 200–250 g (*n* = 6) were exploited. Rats were maintained in a 23 °C, 50% humidity environment under regular light/dark cycles (12 h light:12 h dark) with full access to food and fluid. All operations were performed as directed by the Medical Sciences Guidelines for Animal Handling at Central South University. The study was carried out according to the Ethics Committee’s Guidelines for laboratory animals of Central South University.

After a 1-week acclimatization, rats were allocated into six groups randomly: normal control, BPH model, BPH + Lv-sh-NC, BPH + Lv-sh-METTL3, BPH + Lv-sh-PTEN, and BPH + Lv-sh-METTL3 + Lv-sh-PTEN. Rats in the BPH groups were administered daily with subcutaneous injections of testosterone (5 mg/kg body weight) for 2 weeks. Rats in the normal control group were given a subcutaneous injection of corn oil of equivalent volume. Gross pathologic assessment and comparison of sonographic pictures were exploited to confirm BPH induction. Rats in the BPH + Lv-sh-NC, BPH + Lv-sh-METTL3, BPH + Lv-sh-PTEN, and BPH + Lv-sh-METTL3 + Lv-sh-PTEN groups received corresponding lentivirus injection 2 weeks following the testosterone injection.

At the end of the experiment, overnight-fasted rats were anesthetized with an intraperitoneal administration of pentobarbital sodium solution (100 mg/kg body weight) and sacrificed for tissue collection. The epithelial thickness and the weight of the prostate were evaluated, each rat’s ventral prostate was preserved in 10% neutral-buffered formalin overnight, and the remaining prostate was snap-frozen in liquid nitrogen for mRNA assays.

### m^6^A RNA methylation modification status in BPH tissues

m^6^A in RNA was evaluated with an m^6^A RNA Methylation Assay Kit (Colorimetric) (ab185912, Abcam, Cambridge, MA, USA). Prostate tissues were collected from the normal control and BPH model rats under anesthesia. Total RNA was isolated from target tissues or cells with TRIzol (Invitrogen, CA, USA), and NanoDrop (Thermo Fisher Scientific, Waltham, MA, USA) was employed to determine RNA quality. In general, 200 ng RNA accompanied with m^6^A standard were coated on assay wells. Next, the capture antibody solution and detection antibody solutions were added to assay wells. The quantification of m^6^A levels was conducted by detecting absorbance at 450 nm (OD450) in each well and then calculating relative values using the standard curve.

### qRT-PCR

Total RNA was extracted from target tissues or cells using Trizol Reagent (Invitrogen). cDNA was obtained using the PrimeScriptTM RT reagent kit (Takara, Dalian, China). qRT-PCR was conducted using an SYBR Premix Ex TaqTM kit (Takara Bio) on an ABI 7900HT Real-Time PCR system (Applied Biosystems Life Technologies, Foster City, CA, USA). All final data were evaluated, and the relative expression levels were calculated using the 2^−ΔΔCt^ method. The primer sequence was listed in Table S1.

### Hematoxylin and eosin (H&E) staining

Prostate tissues collected from all groups were H&E stained (Sigma) and evaluated at ×100 magnification using an Olympus microscope (Kyoto, Japan). The existence and extent of epithelial hyperplasia (glandular hyperplasia), stromal edema, inflammation, and degrees were assessed histopathologically in each group by two trial pathologists blinded to the treatment and experimental design.

### Immunohistochemical (IHC) staining

Paraffin-embedded prostate tissues were dissected into 4-μm slices. For antigen retrieval, after deparaffinization in xylene, sections were rehydrated through a descending alcohol row for 3 min each time in 100%, 90%, 80%, 75% ethanol, and then microwaved with sodium citrate buffer, followed by blockage with 5% BSA and treatment overnight at 4 °C with anti-Ki67 (27309-1-AP, Proteintech, Wuhan, China), PCNA (10205-2-AP, Proteintech), E-cadherin (BF0219, Affinity, Cincinnati, OH, USA), N-cadherin (AF4039, Affinity), PTEN (22034-1-AP, Proteintech). Next, sections were incubated at ambient temperature for 60 min with a horseradish peroxidase (HRP)-linked secondary antibody (Proteintech), followed by 3,3′-diaminobenzidine development (DAB Substrate Chromogen System; Dako, Denmark) and hematoxylin staining. An inverted microscope was applied to take images after the slices were fixed (Olympus).

### TUNEL assay detecting apoptosis in tissue samples

TUNEL labeling was performed on paraffin-embedded slices (5-μm thick) after utilizing a colorimetric TUNEL apoptosis assay kit (Beyotime, China) to identify the apoptosis of prostatic epithelial cells. Positive cells with apoptotic nuclei were shown to be brown. After deparaffinization and dehydration by increasing concentrations of ethanol and xylene, the prostate tissue slices were digested in 20 mg/mL proteinase K solution and kept for 15 min at ambient temperature. Reactions were completed through rinsing and incubating sections for 1 h at 37 °C in the Biotin-dUTP TUNEL reaction mixture at dark. Then, sections were further incubated with streptavidin-HRP and DAB staining reagents. Lastly, an optical microscope was employed to analyze mounted sections upon slides.

### Cell lineage, cell culture, and TGF-β stimulation

A human BPH cell line BPH-1 was procured from Merck (SCC256) and cultivated in RPMI 1640 (Gibco, Waltham, MA, USA) containing 20% FBS (Invitrogen, Waltham, MA, USA) at 37 °C in a 5% CO_2_ atmosphere. BPH-1 cells were stimulated with TGF-β (10 ng/ml) for 24 h following the method described previously [52].

### Cell transduction

Silencing of METTL3 was achieved by transducing lentivirus containing short hairpin RNA targeting METTL3 (Lv-sh-METTL3, GenePharma, Shanghai, China). METTL3 overexpression was achieved by transducing plasmid overexpressing METTL3 (METTL3 OE, GenePharma). Silencing of PTEN was achieved by transducing lentivirus containing short hairpin RNA targeting PTEN (Lv-sh-PTEN, GenePharma, Shanghai, China). YTHDF2 silencing or overexpression was achieved by transducing sh-YTHDF2 or YTHDF2 OE vector (GenePharma). Scramble sequence shRNA or empty plasmid was used as negative control (sh-NC/vector). Cell transduction was performed using polybrene (Beyotime) or Lipofectamine 3000 (Invitrogen).

### MTT assay detecting cell viability

Cells (2.5 × 10^3^ cells), transduced and/or treated, were sown in each well of 96-well plates. The cells were then treated with 3-(4, 5-dimethylthiazol-2-yl)-2, 5-diphynyl-2H-tetrazolium bromide (MTT) dye (5 mg/mL) and kept at 37 °C for 4 h. The supernatants were removed, and the formazan crystals were dissolved by adding 100 μL of dimethyl sulfoxide. A microplate reader with a 490 nm wavelength was used to test the violet solution absorbance.

### EdU assay detecting DNA synthesis

Cells, transduced and/or treated, were placed on sterile coverslips in 24-well plates. An EdU Appollo643 kit (RiboBio) was used to measure cell proliferative potential following the product’s protocols, and the nuclei were doubly dyed with EdU and 4’,6-diamidino-2-phenylindole (Beyotime, Shanghai, China). Images were obtained using an inflorescent microscope (Olympus).

### Immunoblotting

Total proteins were collected from target cells with a Cell Mitochondria Isolation Kit (Beyotime, Shanghai, China). The samples were incubated at 4 °C for one night with the primary antibodies METTL3 (15073-1-AP, Proteintech), Bcl-2 (ab196495, Abcam), Bax (50599-2-Ig, Proteintech), Caspase 9 (10380-1-AP, Proteintech), Caspase 3 (19677-1-AP, Proteintech), cleaved PARP-1 (ab32064, Abcam), E-cadherin (BF0219, Affinity), N-cadherin (AF4039, Affinity), vimentin (10366-1-AP, Proteintech), PTEN (22034-1-AP, Proteintech), YTHDF2 (ab220163, Abcam), p53 (CSB-PA07889A0Rb, CUSABIO Life Sciences, College Park, USA), c-Myc (10057-1-AP, Proteintech), NF-κb (ab76302, Abcam), Zeb1 (ab180905, Abcam), Zeb2 (ab191364, Abcam), Snail (ab180714, Abcam), Twist1 (ab50887, Abcam); after that, the samples were incubated at room temperature for 1 h with goat anti-rabbit IgG polyclonal antibody (Abcam) or goat anti-mouse IgG polyclonal antibody (Abcam). GAPDH levels were used as an endogenous control for visualization employing the enhanced chemiluminescence (ECL) detection method (Thermo Fisher Scientific).

### Flow cytometry for cell apoptosis

An annexin V-FITC/PI apoptosis detection kit was used for apoptosis determination. Cells, transduced and/or treated, were collected and suspended in 500 μl binding buffer and incubated with 5 μl Annexin V-FITC and 5 μl PI for 10 min at room temperature at dark. Then cells were evaluated using Flow cytometry (Novocyte, Agilent, USA).

### MeRIP sequencing and RNA sequencing

Total RNA from each sample was isolated using TRIzol reagent (Invitrogen) and then quantified using a NanoDrop ND-1000 instrument. mRNA was isolated first from total RNA samples using the Arraystar Seq-StarTM poly(A) mRNA Isolation Kit and then fragmented into 100-nucleotide-long fragments. The size of fragmented mRNAs was confirmed using agarose electrophoresis. m^6^A-methylated mRNA fragments were enriched by immunoprecipitation with an anti-m^6^A antibody (Synaptic Systems, 202003). A portion of the original fragmented mRNAs was kept as input. RNA-seq libraries of m^6^A antibody-enriched mRNAs and input mRNAs were prepared using the KAPA Stranded mRNA-seq Kit (Illumina) as follows: (1) first-strand cDNA synthesis; (2) second-strand cDNA synthesis; (3) A-tailing; (4) adapter ligation; (5) PCR amplification; and (6) library purification. Quality control of the completed libraries was performed with an Agilent 2100 Bioanalyzer. Libraries were mixed in equal amounts according to the quantification results by qPCR and used for sequencing on the instrument. The DNA fragments in well-mixed libraries were denatured with 0.1 M NaOH to generate single-stranded DNA molecules and loaded onto the reagent cartridge at the appropriate loading concentration. Clustered libraries were then sequenced on the Illumina NovaSeq 6000 following the NovaSeq 6000 S4 Reagent Kit (300 cycles) protocol.

### MeRIP-qPCR

Magna MeRIP m^6^A kit (17–10,499, Millipore) was employed to perform m^6^A RNA immunoprecipitation (MeRIP) as directed by the manufacturer’s protocol. The IP production was conducted RT-qPCR for PTEN as mentioned above. Primers used for RT-qPCR are presented in Table S1.

### Actinomycin D detecting mRNA stabilization

Cells were incubated with 5 μg/ml actinomycin D for 0, 6, and 12 h, and the relative levels of target mRNA were quantified by performing qPCR assays. Target mRNA degradation rate was calculated as directed by a previously reported study [53].

### RIP

Cells were washed by cold PBS and lysed using RIP lysis buffer (300 mM NaCl, 0.2% NP-40, 20 mM Tris-HCl PH 7.6, 0.5 mM DTT, protease inhibitor, and RNase inhibitor) at 4 °C via disruptive sonication. Next, the lysis was incubated at 4 °C overnight with 5 μg anti-YTHDF2 Rabbit antibody, or IgG (ProteinTech Group) pre-conjugated protein A/G Magnetic Beads (Millipore) in 500 μl IP buffer (150 mM NaCl, 10 mM Tris-HCl (pH 7.4), 1 mM EDTA, 1 mM EGTA, 1% Triton X-100, 0.5% NP-40) containing RNase inhibitors (Thermo Fisher). The IP complex was treated for 30 min at 55 °C with Proteinase K (Thermo Fisher), and RNA was purified using phenol:chloroform:isoamyl alcohol. Last, RT-qPCR was carried out to determine PTEN mRNA levels in immunoprecipitate by anti-IgG or anti-YTHDF2. Primers used for RT-qPCR are presented in Table S1.

### Statistical analysis

GraphPad software (San Diego, CA, USA) was employed to process data from at least three independent experiments. All data were presented as the means ± standard deviation (SD). One-way analysis of variance was employed, followed by Tukey’s multiple comparison test or independent sample *t* test. The significance level was set at *P* < 0.05.

## Supplementary information

Supplemental data

Original Data File

A reproducibility checklist

## Data Availability

The authors confirm that the data supporting the findings of this study are available within the article.
