# Supplementary material for: METTL3 promotes prostatic hyperplasia by regulating PTEN expression in an m6A-YTHDF2-dependent manner
Source: Cell Death Dis. 2022 Aug 19;13(8):723. doi: 10.1038/s41419-022-05162-4 (PMC9391461; doi:10.1038/s41419-022-05162-4)
Supplement: Supplementary file 1 — Supplemental data [file 41419_2022_5162_MOESM1_ESM.docx]

[**Supplemental data**](https://www.ncbi.nlm.nih.gov/pmc/articles/PMC8590697/bin/41419_2021_4367_MOESM1_ESM.docx)


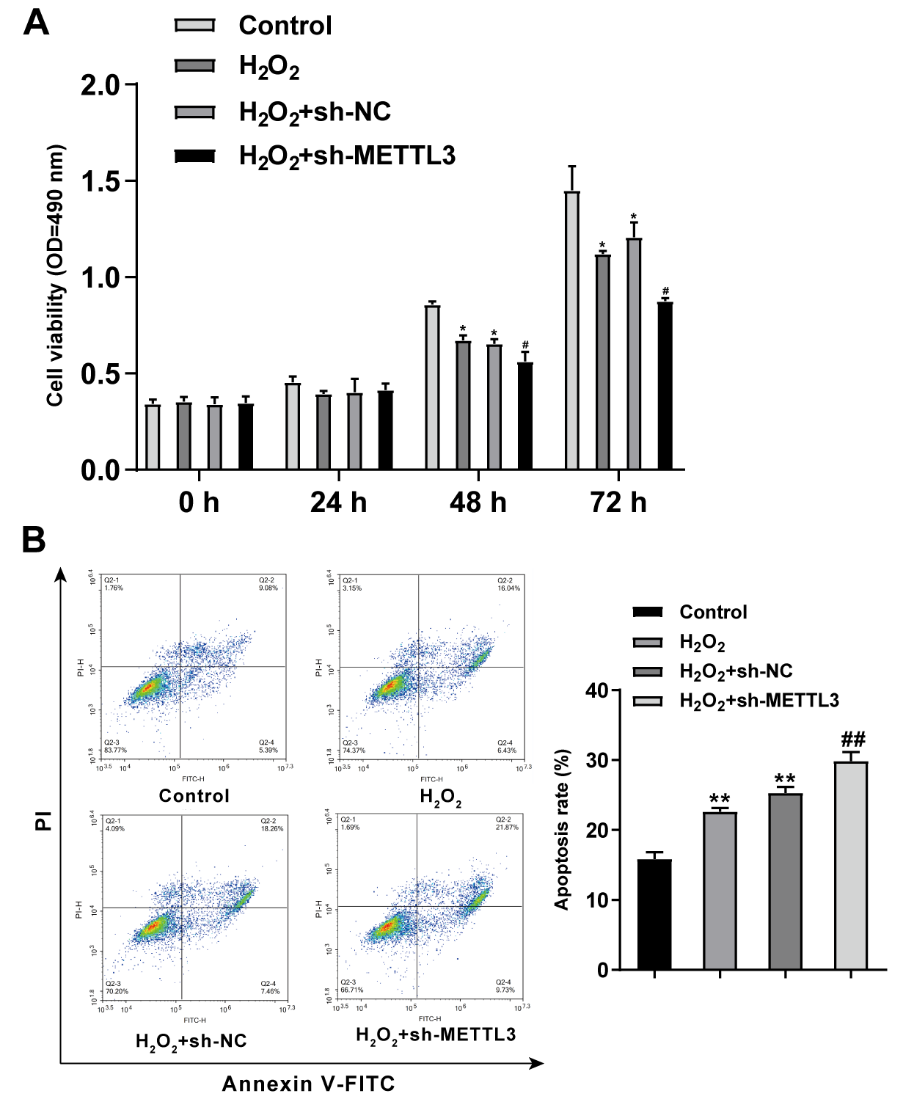


**Fig.S1 The effects of METTL3 knockdown on the cellular survival and apoptosis of the BPH-1 cells under cytotoxic conditions** BPH-1 cells were transduced with sh-METTL3 for 24 h then further stimulated with H_2_O_2_ (150 μM, 6 h). (A) The cell viability of BPH-1 cells in each group were detected using MTT assay. (B) The cell apoptosis of BPH-1 cells in each group were detected using Flow cytometry. **P*<0.05, ***P*<0.01, compared with the control group; #*P*<0.05, ##*P*<0.01, compared with the H_2_O_2_+sh-NC group.


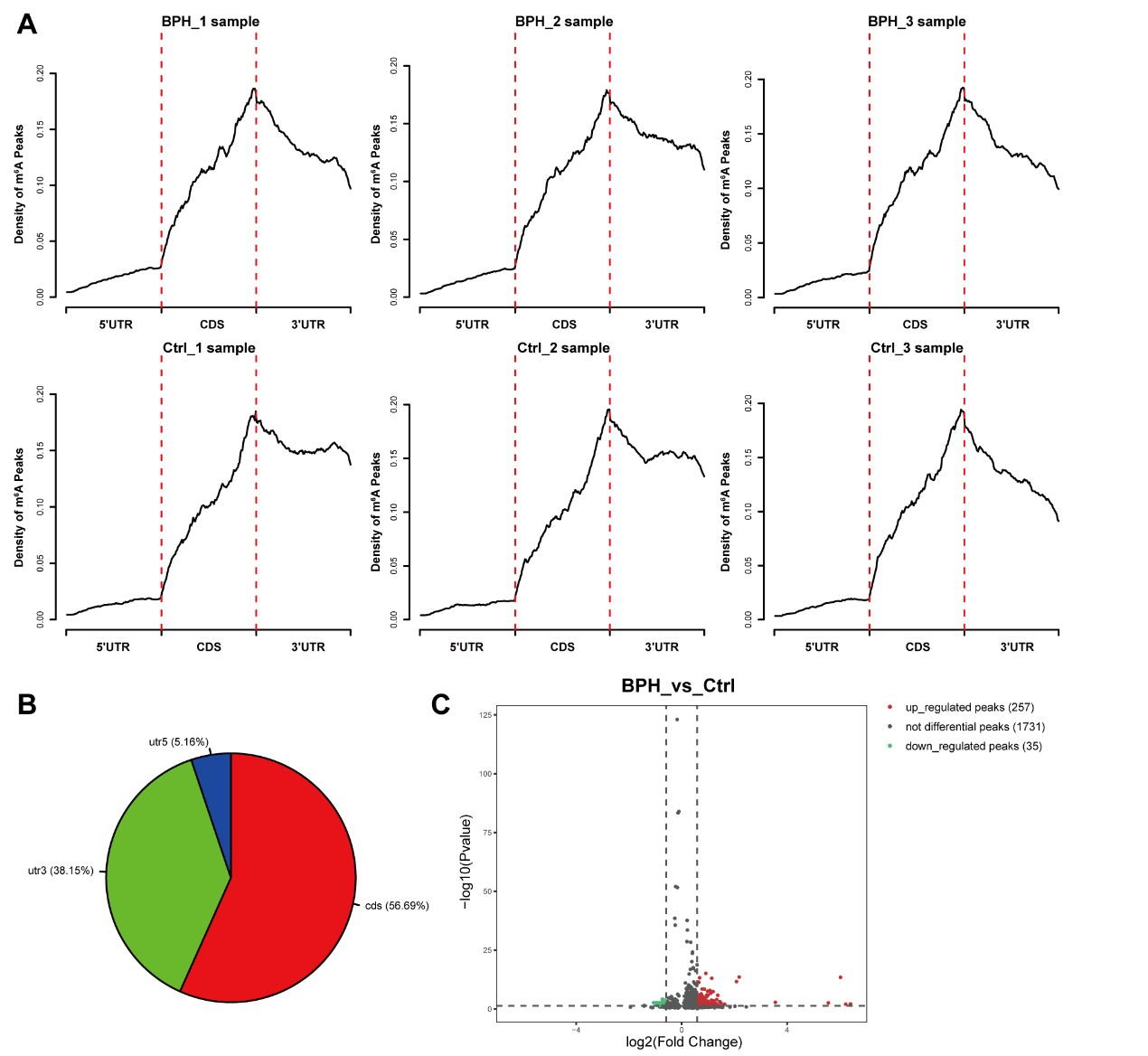


**Fig.S2 m^6^A sequencing analysis of BPH rats** (A-B) Map and percentage of m^6^A modification locations in chromosomal regions. (C) Analysis of differential m^6^A loci.


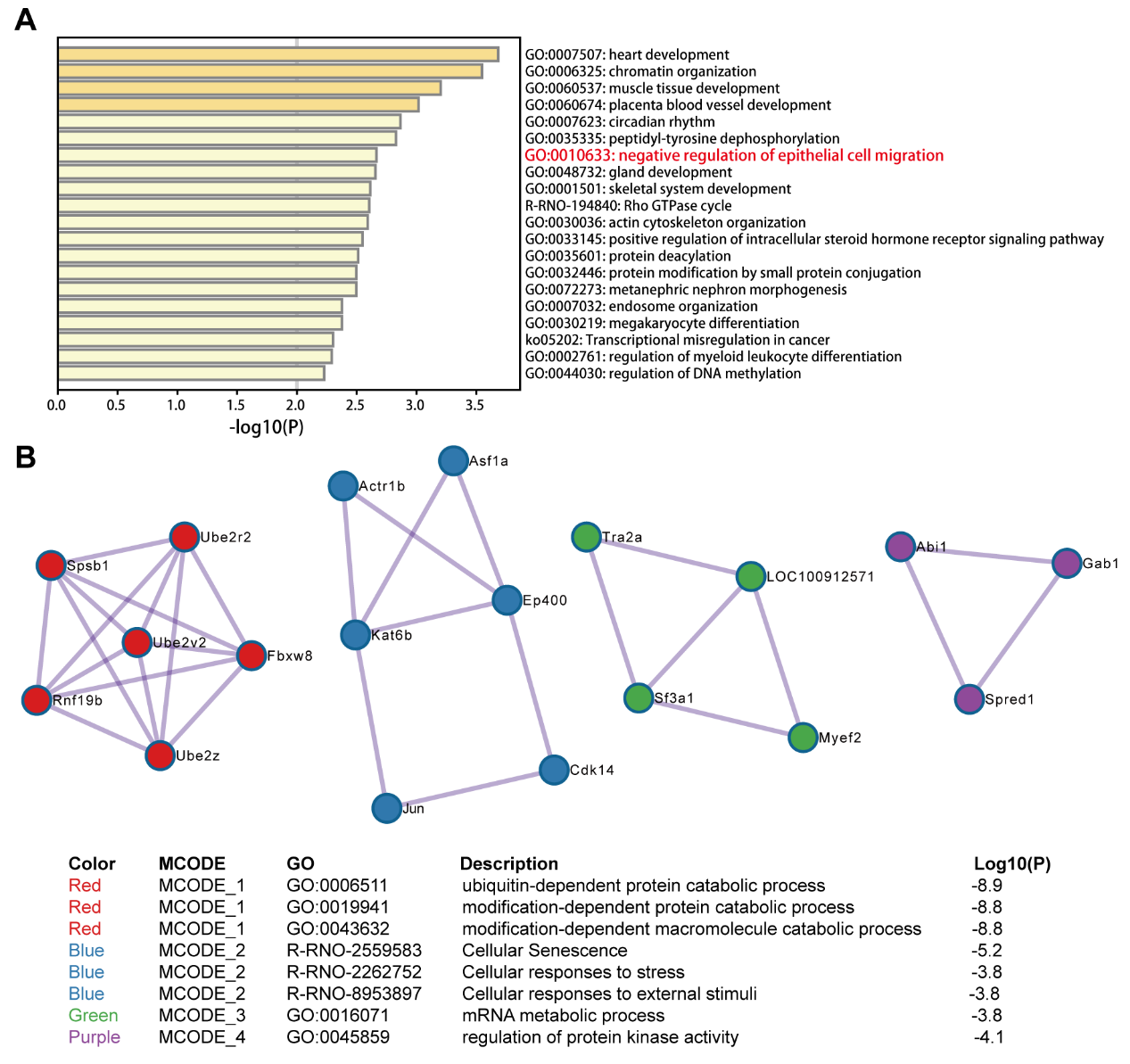


**Fig.S3 Functional enrichment annotation on differentially expressed genes** (A) Functional enrichment annotation analysis of 241 up-regulated and down-regulated genes using Metascape (<https://metascape.org/>). (B) Protein interaction network and MCODE module analysis.


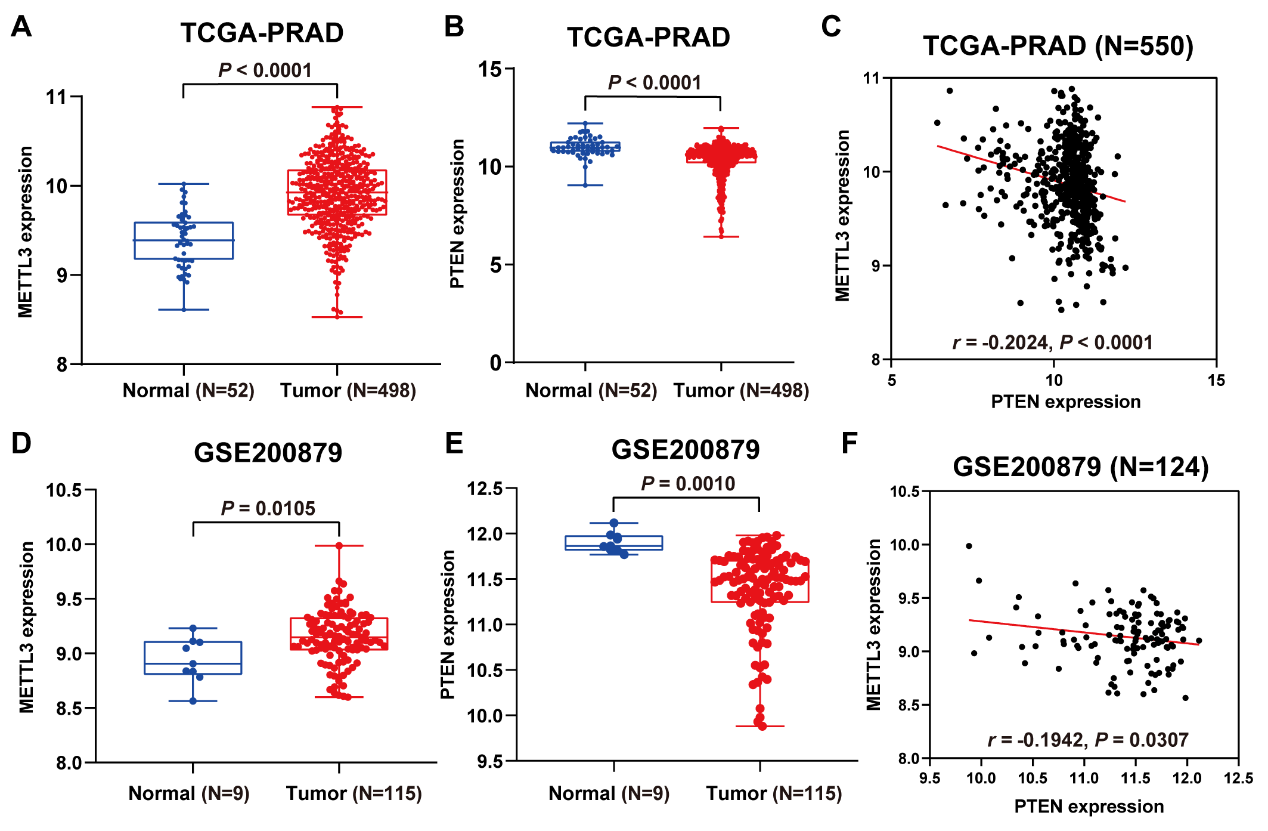


**Fig.S4 The METTL3 and PTEN expression and their correlation in prostatic carcinoma samples** (A-B) METTL3 and PTEN expressions were analyzed in normal control (N=52) and prostatic carcinoma (N=498) samples according to The Cancer Genome Atlas Prostate Adenocarcinoma (TCGA-PRAD) dataset. (C) The correlation between METTL3 and PTEN expression was analyzed using Pearson’s correlation analysis based on TCGA-PRAD. (D-E) METTL3 and PTEN expressions were analyzed in normal control (N=9) and prostatic carcinoma (N=115) samples according to Gene Expression Omnibus (GEO) dataset (GSE200879). (F) The correlation between METTL3 and PTEN expression was analyzed using Pearson’s correlation analysis based on GSE200879.

**
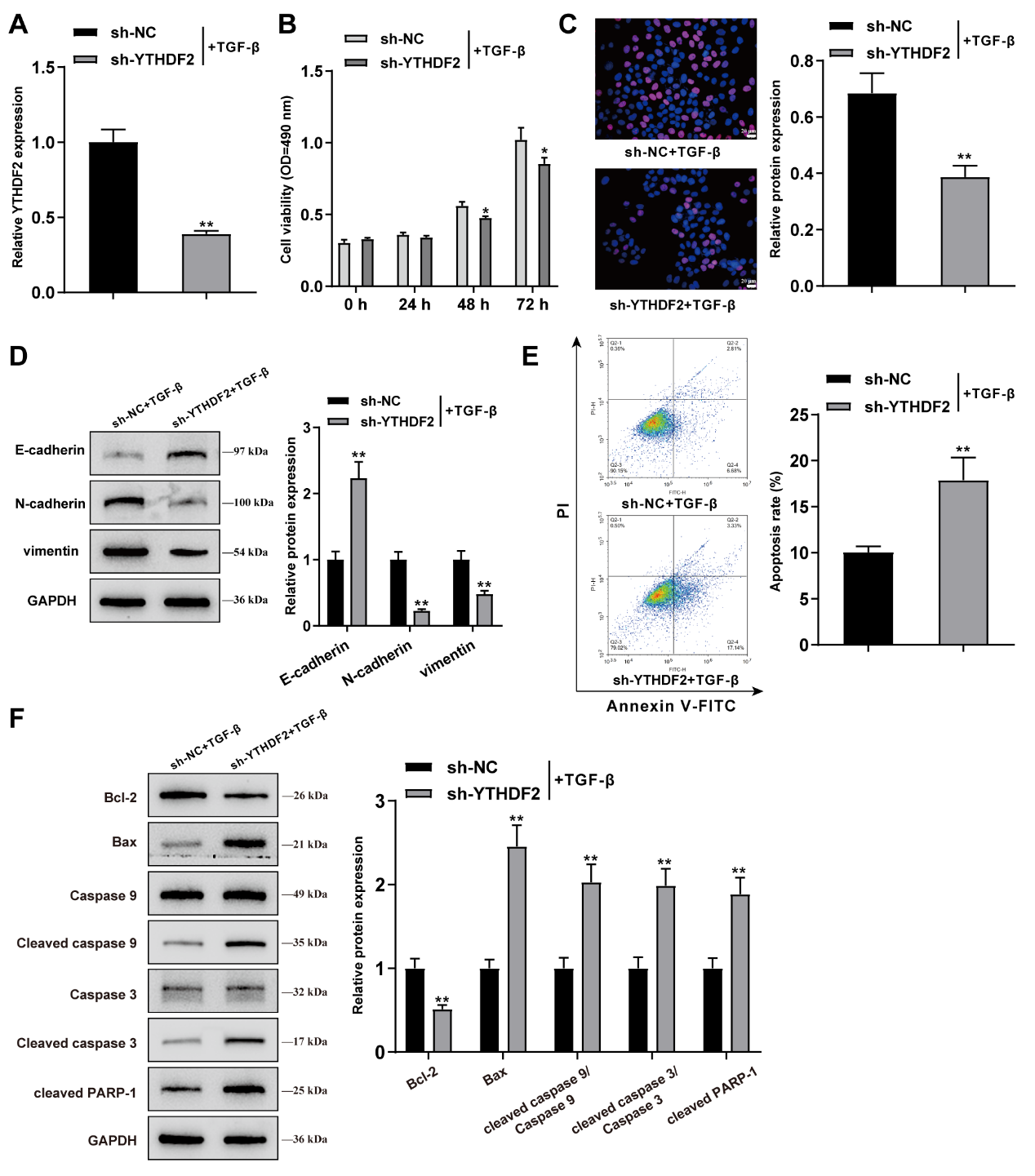
**

**Fig.S5 Effects of YTHDF2 knockdown on proliferation, apoptosis and EMT of TGF-β-treated BPH-1 cells** YTHDF2 knockdown was achieved in TGF-β-treated BPH-1 cells by transducing sh-YTHDF2 and then examined for (A) YTHDF2 expression using qRT-PCR; (B) cell viability using MTT assay; (C) DNA synthesis using EdU assay; Scale bar = 20 μm; (D) the protein levels of E-cadherin, N-cadherin, vimentin using Immunoblotting; (E) cell apoptosis using Flow cytometry; (F) the protein levels of Bcl-2, Bax, Caspase 9, cleaved caspase 9, Caspase 3, cleaved caspase 3, and cleaved PARP-1 using Immunoblotting. **P*<0.05, ***P*<0.01, compared with the sh-NC group.


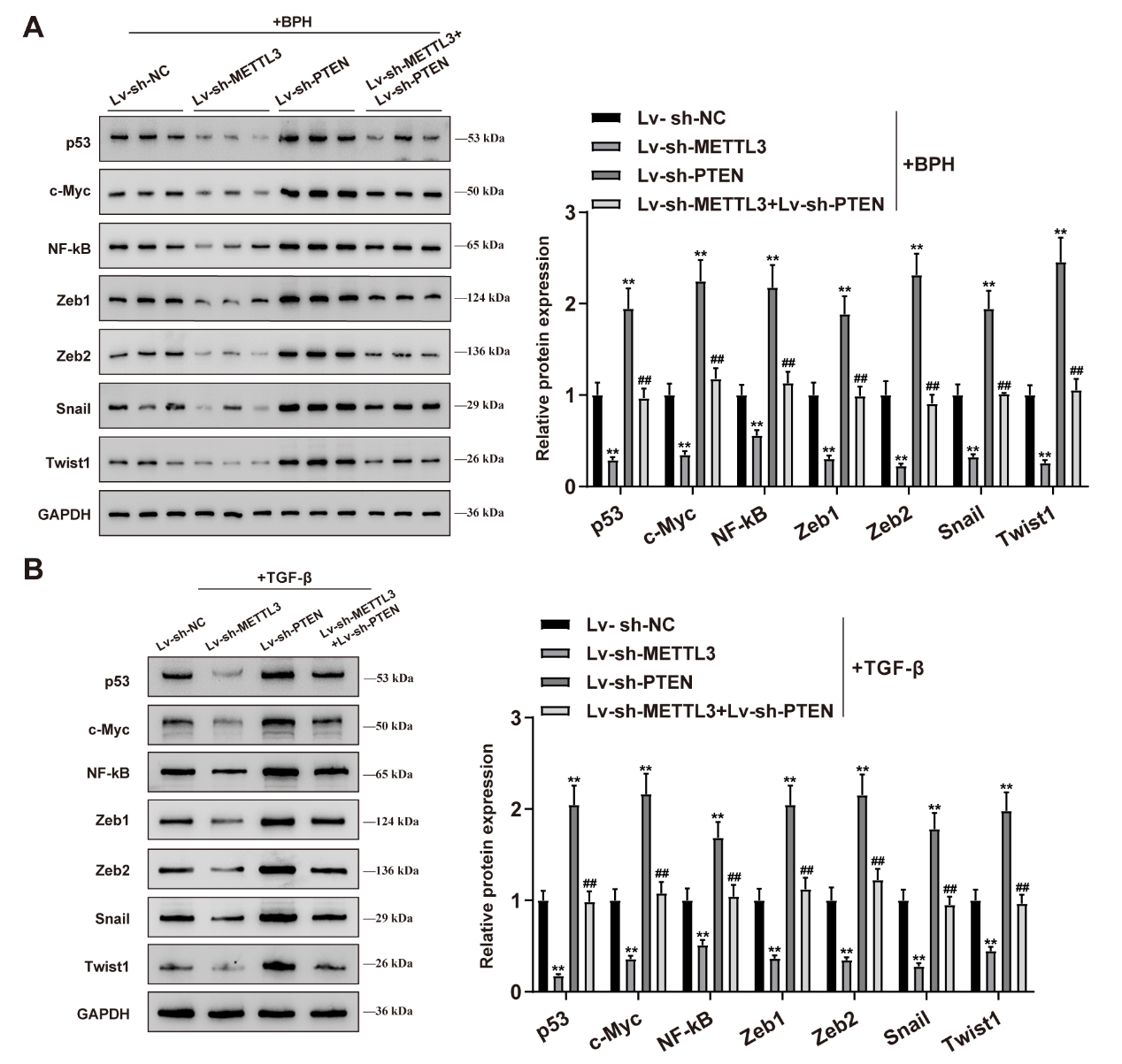


**Fig.S6 METTL3 regulates PTEN and affects the proliferation, EMT, and apoptosis transcription factors** (A) The protein levels of proliferation, EMT, and apoptosis transcription factors (p53, c-Myc, NF-κB, Zeb1, Zeb2, Snail, Twist1) in BPH rats' prostate were determined using Immunoblotting. (B) The protein levels of proliferation, EMT, and apoptosis transcription factors (p53, c-Myc, NF-κB, Zeb1, Zeb2, Snail, Twist1) in TGF-β-stimulated BPH-1 cells were determined using Immunoblotting. ***P*<0.01, compared with the Lv-sh-NC group. ##*P*<0.01, compared with the Lv-sh-METTL3 group.

**Table S1. The primer sequence for the study**

|  | **Forward (5’-3’)** | **Reverse (5’-3’)** |
| --- | --- | --- |
| RT-PCR  METTL3 (Rattus norvegicus) | CAAGCACACTGATGAGTCTTTAGGT | ACTCTCAGAATCCACACAGGCG |
| RT-PCR PTEN  (Rattus norvegicus) | CGTGCGGATAATGACAAGGA | ATTTGATGGCTCCTCTACTGTTTTT |
| RT-PCR  YTHDF2 (Rattus norvegicus) | ACTCAGACATCAGTAGGGCAACAGA | CCACCCCATTATGACCGAACC |
| RT-PCR  METTL14 (Rattus norvegicus) | TGAAGCGAAGCACAGATGGG | GTGAAAAATCTCCACGGGCTT |
| RT-PCR  WTAP (Rattus norvegicus) | AAAGTTATGGCACGGGATGAGT | TGCTGTTGCTGCTTTAGTTTTTCT |
| RT-PCR  FTO (Rattus norvegicus) | GTGTCTCGCATCCTCATTGGG | GCCTTCTCTTTGATAGCCAGTTCTT |
| RT-PCR  ALKBH5 (Rattus norvegicus) | CTTTCTTTAGCGACTCGGCACTT | CCTGAGAATGATGACCGCCC |
| RT-PCR  YTHDF1 (Rattus norvegicus) | ACCCTCCCATCCCGTATCTCACTA | CTGTGCTGGTAAATGTTGTTCCCC |
| RT-PCR  YTHDC2 (Rattus norvegicus) | CCCATCCTTACAATCGCCTGCA | GGCTCGCTCCCACCCATCAC |
| RT-PCR  GAPDH (Rattus norvegicus) | GCCTTCCGTGTTCCTACCCC | CGCCTGCTTCACCACCTTCT |
| RT-PCR  METTL3 (Homo sapiens) | CATTGCCCACTGATGCTGTG | AGGCTTTCTACCCCATCTTGA |
| RT-PCR  YTHDF2 (Homo sapiens) | CCTTAGGTGGAGCCATGATTG | TCTGTGCTACCCAACTTCAGT |
| RT-PCR/RIP-PCR  PTEN (Homo sapiens) | TGAAGACCATAACCCACCACAGC | TACACCAGTTCGTCCCTTTCCAG |
| MERIP-qPCR  PTEN 3’UTR (Homo sapiens) | CTAAGAGAGGTTTCCGAAGGGTT | GTGTGTGTAAATAGCTGGAGATGGT |
| RT-PCR  GAPDH (Homo sapiens) | ACAGCCTCAAGATCATCAGC | GGTCATGAGTCCTTCCACGAT |
| Lv-sh-NC | GATCCGCAGATGAAGGCACGGTCACGCTCGAGGCAGATGAAGGCACGGTCACGTTTTTG | AATTCAAAAAGCAGATGAAGGCACGGTCACGCTCGAGGCAGATGAAGGCACGGTCACGG |
| Lv-sh-METTL3 | GATCCCCTTCTACAGAAATTTGCTGCCTCGAGGCAGCAAATTTCTGTAGAAGGTTTTTG | AATTCAAAAACCTTCTACAGAAATTTGCTGCCTCGAGGCAGCAAATTTCTGTAGAAGGG |
| Lv-METTL3 OE | CTACCGGACTCAGATCTCGAGATGTCGGACACGTGGAGCTC | GTACCGTCGACTGCAGAATTCCTATAAGTTCTTAGGTTTAGAGATGACACCA |
| Lv-sh-YTHDF2 | GATCCGATTTCGAACCTTACTTGAGCCTCGAGGCTCAAGTAAGGTTCGAAATCTTTTTG | AATTCAAAAAGATTTCGAACCTTACTTGAGCCTCGAGGCTCAAGTAAGGTTCGAAATCG |
| Lv-YTHDF2 OE | CTACCGGACTCAGATCTCGAGATGTCGGCCAGCAGCCTC | GTACCGTCGACTGCAGAATTCTTATTTCCCACGGCCTTGAC |
| Lv-sh-PTEN | GATCCGGGTAAATACGTTCTTCATACCTCGAGGTATGAAGAACGTATTTACCCTTTTTG | AATTCAAAAAGGGTAAATACGTTCTTCATACCTCGAGGTATGAAGAACGTATTTACCCG |
