# Supplementary material for: METTL3 promotes prostatic hyperplasia by regulating PTEN expression in an m6A-YTHDF2-dependent manner
Source: Cell Death Dis. 2022 Aug 19;13(8):723. doi: 10.1038/s41419-022-05162-4 (PMC9391461; doi:10.1038/s41419-022-05162-4)
Supplement: Supplementary file 2 — Original Data File [file 41419_2022_5162_MOESM2_ESM.docx]

**Original western blot images**

Figure 1D-METTL3

**

**

Figure 1D-GAPDH

**

**

Figure 2E-Bcl-2





Figure 2E-Bax





Figure 2E-Caspase 9





Figure 2E-Cleaved caspase 9





Figure 2E-Caspase 3





Figure 2E-Cleaved caspase 3





Figure 2E-cleaved PARP-1


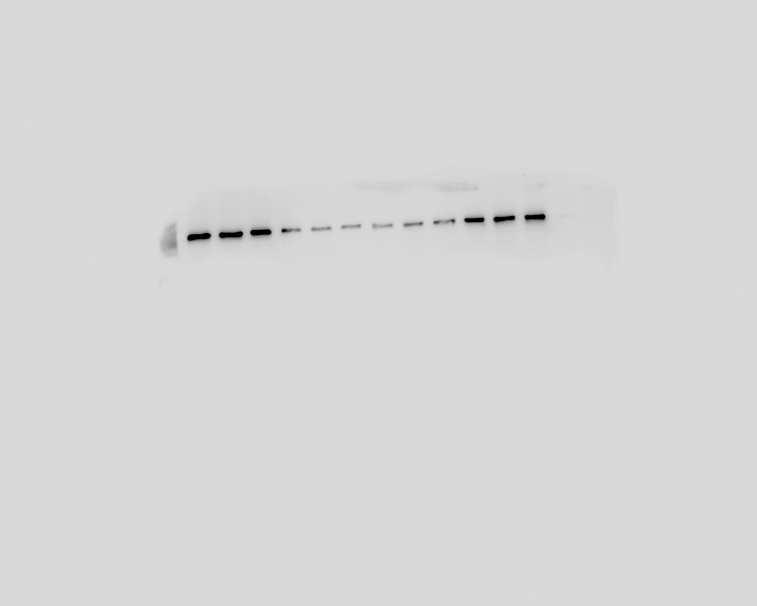


Figure 2E-GAPDH





Figure 3F-E-cadherin





Figure 3F-N-cadherin





Figure 3F-Vimentin





Figure 3F-GAPDH





Figure 3H-Bcl-2





Figure 3H-Bax





Figure 3H-Caspase 9





Figure 3H-Cleaved caspase 9





Figure 3H-Caspase 3





Figure 3H-Cleaved caspase 3





Figure 3H-Cleaved PARP-1





Figure 3H-GAPDH





Figure 4F-PTEN





Figure 4F-GAPDH





Figure 5C-PTEN





Figure 5C-GAPDH





Figure 5I-YTHDF2


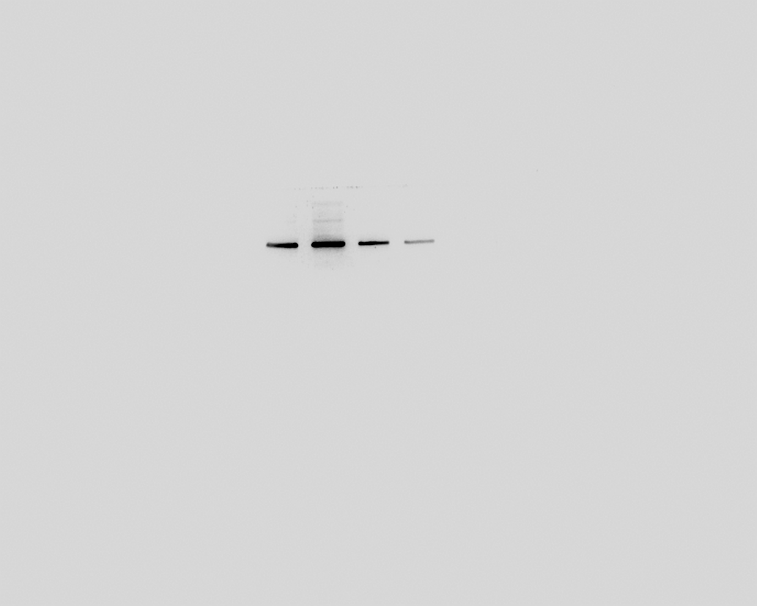


Figure 5I-GAPDH





Figure 5K-PTEN





Figure 5K-GAPDH





Figure 6B-METTL3





Figure 6B-GAPDH-1





Figure 6B-PTEN





Figure 6B-GAPDH-2





Figure 7E-E-cadherin





Figure 7E-N-cadherin





Figure 7E-Vimentin





Figure 7E-GAPDH





Figure S5D-E-cadherin





Figure S5D-N-cadherin





Figure S5D-Vimentin


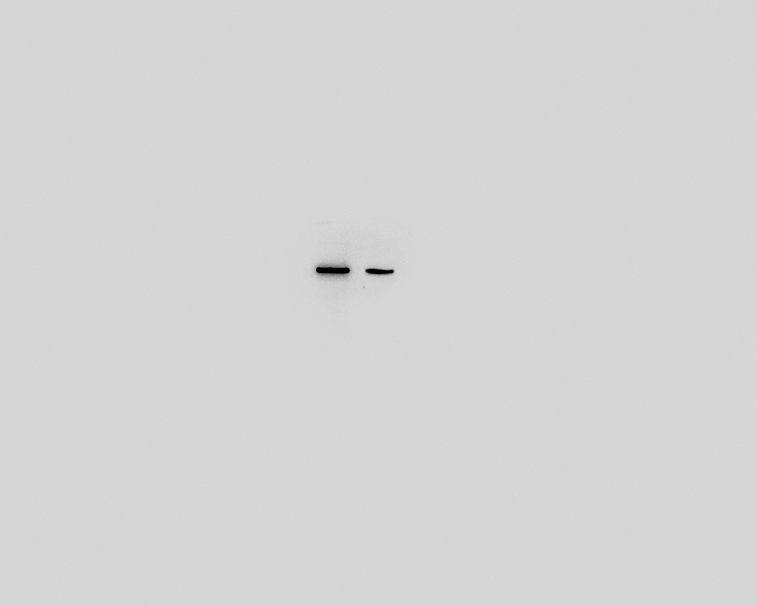


Figure S5D-GAPDH





Figure S5F-Bcl-2





Figure S5F-Bax





Figure S5F-Caspase 9





Figure S5F-Cleaved caspase 9





Figure S5F-Caspase 3





Figure S5F-Cleaved caspase 3





Figure S5F-Cleaved PARP-1





Figure S5F-GAPDH





Figure S6A-p53





Figure S6A-c-Myc


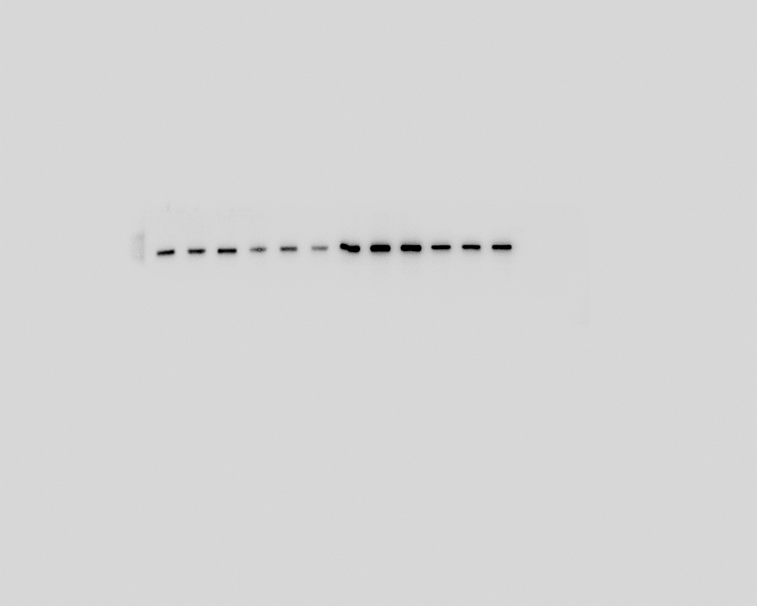


Figure S6A-NF-κB


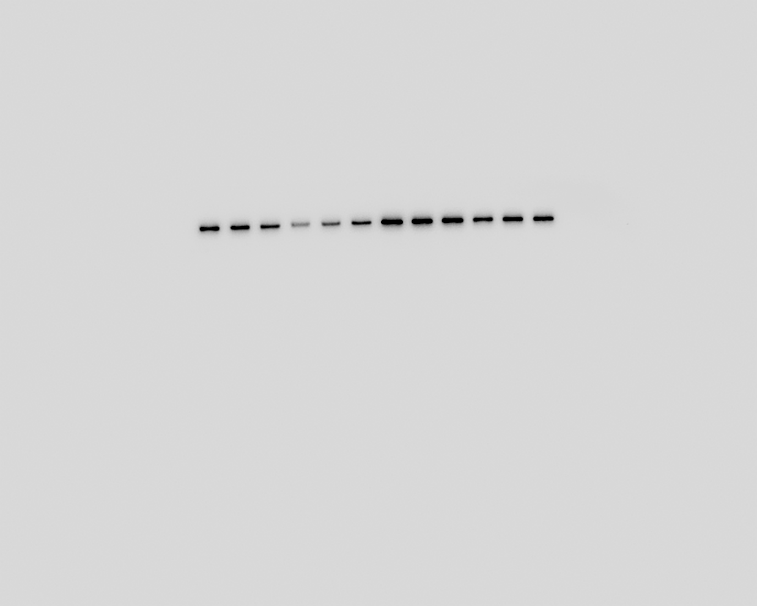


Figure S6A-Zeb1





Figure S6A-Zeb2


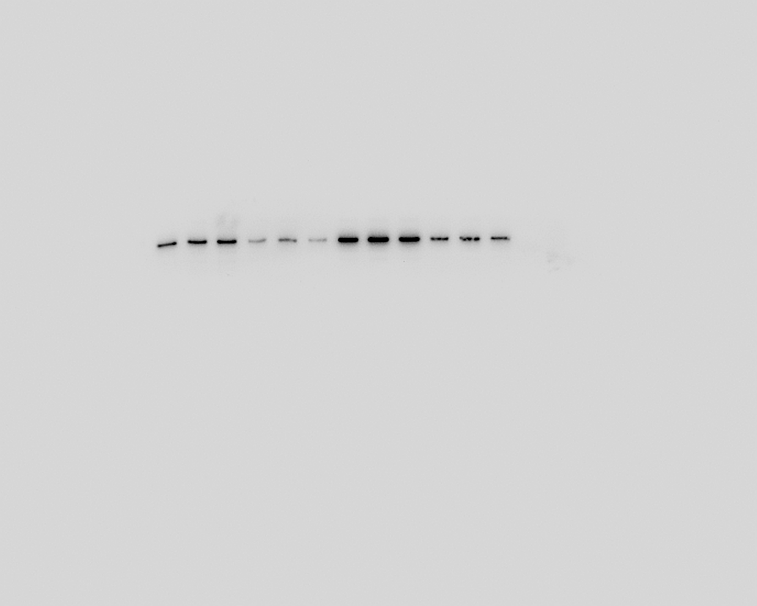


Figure S6A-Snail





Figure S6A-Twist1





Figure S6A-GAPDH





Figure S6B-p53





Figure S6B-c-Myc





Figure S6B-NF-κB





Figure S6B-Zeb1


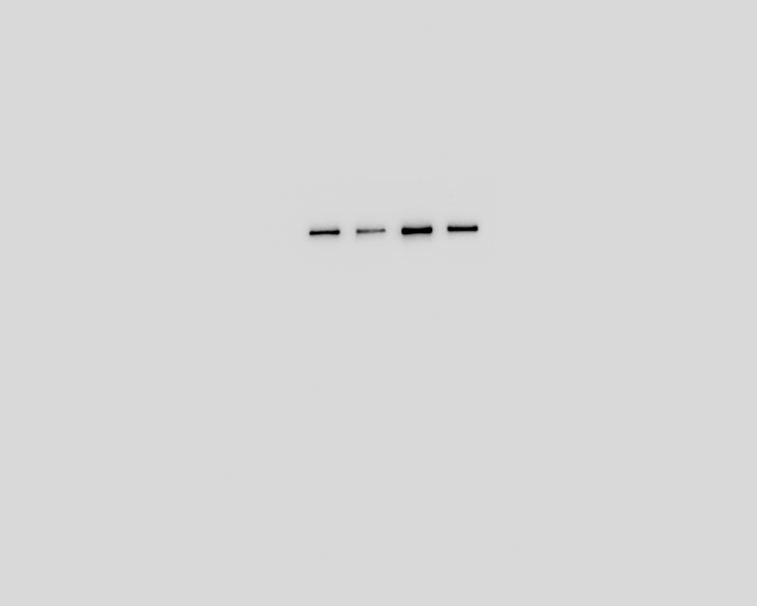


Figure S6B-Zeb2


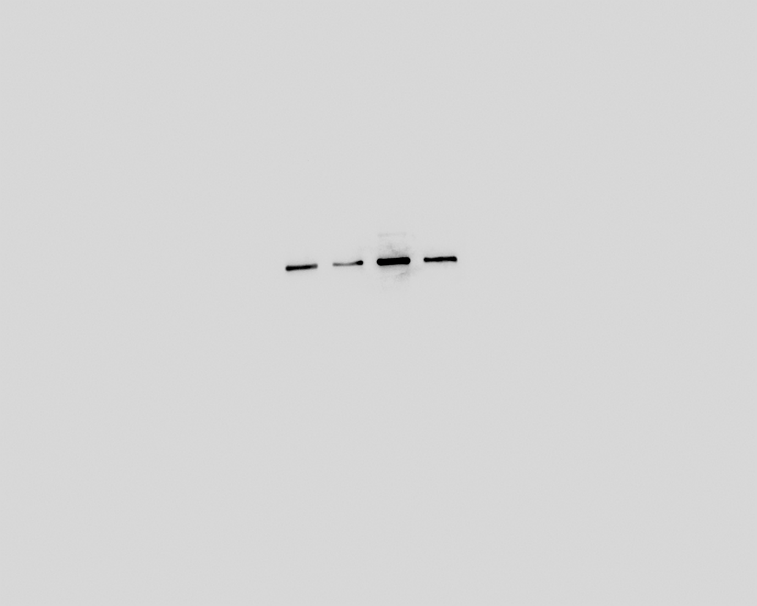


Figure S6B-Snail


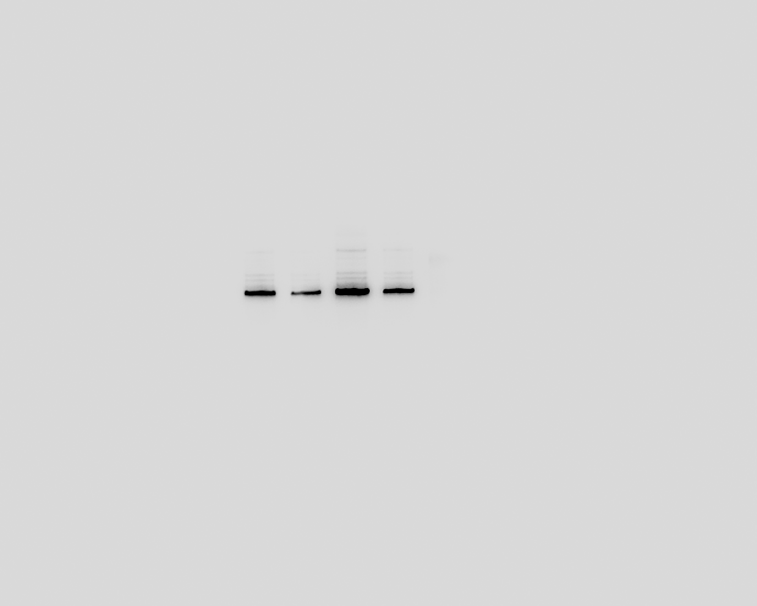


Figure S6B-Twist1





Figure S6B-GAPDH
